# HGF Mediates Clinical‐Grade Human Umbilical Cord‐Derived Mesenchymal Stem Cells Improved Functional Recovery in a Senescence‐Accelerated Mouse Model of Alzheimer's Disease

**DOI:** 10.1002/advs.201903809

**Published:** 2020-07-06

**Authors:** Yali Jia, Ning Cao, Jinglei Zhai, Quan Zeng, Pei Zheng, Ruyu Su, Tuling Liao, Jiajing Liu, Haiyun Pei, Zeng Fan, Junnian Zhou, Jiafei Xi, Lijuan He, Lin Chen, Xue Nan, Wen Yue, Xuetao Pei

**Affiliations:** ^1^ Stem Cell and Regenerative Medicine Lab Institute of Health Service and Transfusion Medicine Beijing 100850 China; ^2^ Experimental Hematology and Biochemistry Lab Beijing Institute of Radiation Medicine Beijing 100850 China; ^3^ South China Institute of Biomedicine Guangzhou 510005 China; ^4^ 920th Hospital of Joint Logistics Support Force Kunming 650032 China

**Keywords:** AKT‐GSK3*β* signaling pathway, Alzheimer's disease, cMet, hepatocyte growth factor, hyperphosphorylated tau, umbilical cord‐derived mesenchymal stem cells

## Abstract

Stem cells have emerged as a potential therapy for a range of neural insults, but their application in Alzheimer's disease (AD) is still limited and the mechanisms underlying the cognitive benefits of stem cells remain to be elucidated. Here, the effects of clinical‐grade human umbilical cord‐derived mesenchymal stem cells (hUC‐MSCs) on the recovery of cognitive ability in SAMP8 mice, a senescence‐accelerated mouse model of AD is explored. A functional assay identifies that the core functional factor hepatocyte growth factor (HGF) secreted from hUC‐MSCs plays critical roles in hUC‐MSC‐modulated recovery of damaged neural cells by down‐regulating hyperphosphorylated tau, reversing spine loss, and promoting synaptic plasticity in an AD cell model. Mechanistically, structural and functional recovery, as well as cognitive enhancements elicited by exposure to hUC‐MSCs, are at least partially mediated by HGF in the AD hippocampus through the activation of the cMet‐AKT‐GSK3*β* signaling pathway. Taken together, these data strongly implicate HGF in mediating hUC‐MSC‐induced improvements in functional recovery in AD models.

## Graphical abstract

This study explores the effects of clinical‐grade hUC‐MSCs on the recovery of cognitive ability of Alzheimer's disease (AD). All results uncover that HGF secreted from hUC‐MSCs, mediates the beneficial effects of hUC‐MSCs on functional recovery of damaged neural cells by downregulating hyperphosphorylated tau, improving neurofibrillary tangles, reversing spine loss, and promoting synaptic plasticity in AD hippocampus through the cMet‐AKT‐GSK3*β* signaling pathway.

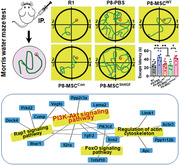

## 1 Introduction

Alzheimer's disease (AD) is a progressive neurodegenerative disorder that is characterized by neuronal loss and cognitive decline. AD is rapidly becoming one of the leading causes of disability and mortality in the elderly.^[^ ^1^, ^2^ ^]^ The brains of patients with AD, in addition to showing nerve and synapse loss, are histopathologically characterized by two hallmark lesions: amyloid‑*β* (A*β*)‑containing plaques and neurofibrillary tangles (NFTs), which are composed of hyperphosphorylated forms of the microtubule‑associated protein tau.^[^ ^3^, ^4^ ^]^ Recently, more new light has been shed on the possible interactions of A*β* and tau, and novel findings have shifted our understanding of the role of tau which acts downstream of A*β* or toward being a crucial partner of A*β* to induce neuronal death in the pathogenesis of AD,^[^ ^5^, ^6^ ^]^ which prompts that targeting tau pathology might be a clinically effective therapy or targeting both tau and A*β* seems prudent. Current treatments for AD are solely symptomatic and pharmacological agents that are unable to attenuate disease progression. Thus, advancing our understanding of AD mechanisms is critical to improve the efficacy of current treatment strategies.

Stem cell‐based therapy has emerged as a promising approach to treat neurodegenerative diseases.^[^ ^7^, ^8^ ^]^ In recent years, accumulating evidence indicates that the transplantation of adult stem cells such as bone marrow stem cells (BMSCs), adipose mesenchymal stem cells (ADMSCs) or neural stem cells (NSCs) into the lateral ventricles ameliorates cognitive deficits in AD animal models.^[^ ^9^, ^10^, ^11^, ^12^, ^13^ ^]^ Nevertheless, many factors limit their clinical applications, including low yield, ethical issues, and invasive procedures. Compared to the above stem cells, umbilical cord‐derived mesenchymal stem cells (hUC‐MSCs) maintain an earlier embryologic phase, are much younger, have a higher yield without ethical issues and invasive procedures, and can secrete a wide range of multifunctional factors; more importantly, only hUC‐MSCs can meet all these advantages at the same time. These factors suggest that hUC‐MSCs may be a better choice for clinical application compared to many other stem cells.

Recently, our group optimized transplantation parameters and established several benefits of hUC‐MSC‐based interventions for normal aging‐related cognitive decline.^[^ ^14^ ^]^ We demonstrated that the intraperitoneal administration of clinical‐grade hUC‐MSCs could counteract cognitive aging of synaptic plasticity, neural networks, molecular regulation, and cognition in aged mice; these effects were systemic and integrated, suggesting that hUC‐MSCs may be a preferred resource to systematically regulate the aging brain and interpose the physiological processes of cognitive aging.^[^ ^14^ ^]^ However, whether the hUC‐MSCs are a better choice for treating pathological cognitive decline in the aging brain, especially for AD, still needs to be further addressed. Therefore, we hypothesized that hUC‐MSCs, as young stem cells,^[^ ^15^ ^]^ may be a superior source for interposing or reversing AD. More importantly, hUC‐MSCs can secrete a wide range of functional factors, including growth factors, cytokines, chemokines, and metabolites; so, there is still a valuable research topic that is which core functional factors secreted from hUC‐MSCs plays important roles in hUC‐MSC‐modulated recovery of cognition function.

Here, for the first time, we explored the effects of clinical‐grade hUC‐MSCs on the recovery of cognitive ability in SAMP8 mice, which is a senescence‐accelerated mouse model of AD; and we demonstrated that the core functional factor, hepatocyte growth factor (HGF), secreted from hUC‐MSCs plays important roles in hUC‐MSC‐modulated recovery of damaged neural cells by down‐regulating hyperphosphorylated tau, improving neurofibrillary tangles, reversing spine loss, and promoting synaptic plasticity in the aged hippocampus, which are all closely associated with memory deficits. Mechanistically, structural and functional recovery, as well as cognitive enhancements elicited by exposure to hUC‐MSCs, were at least partially mediated by HGF in the AD hippocampus through the activation of the cMet‐AKT‐GSK3*β* signaling pathway.

Collectively, we demonstrate that HGF, a pleiotropic cytokine secreted by hUC‐MSCs, mediates the beneficial effects of hUC‐MSCs on functional recovery in AD related models via cMet‐AKT‐GSK3*β* signaling pathway. Our study highlights a promising strategy for AD intervention based on the pleiotropic cytokine HGF, which could be used in isolation or in combination with hUC‐MSCs for AD treatment.

## 2 Results

### 2.1 Clinical‐Grade hUC‐MSCs Improved Spatial Learning Ability and Memory in SAMP8 Mice

**Figure 1:**
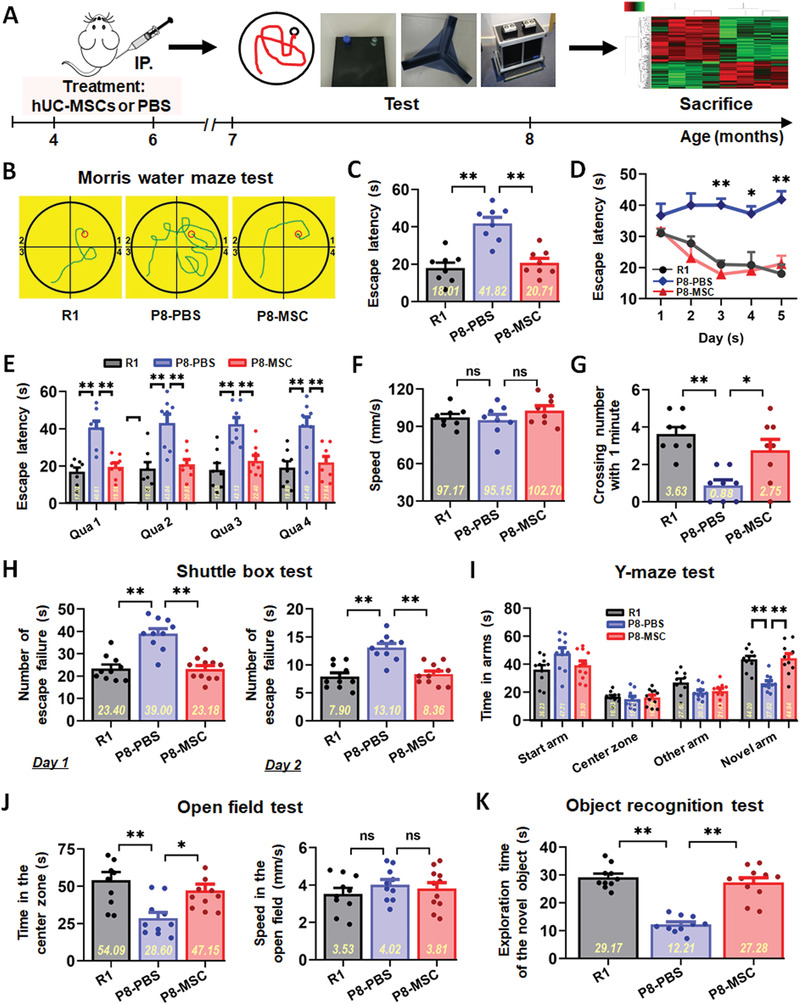
hUC‐MSCs improved spatial learning and memory ability in SAMP8 mice. A) Schematic illustrating the chronological order used for hUC‐MSCs or PBS treatment, Morris water maze (MWM), shuttle box, Y‐maze, open field and object recognition testing. B–G) Results from SAMR1 (R1), SAMP8‐PBS (P8‐PBS), and SAMP8‐MSC (P8‐MSC) groups that were cognitively tested by Morris water maze. The typical escape way (B), the escape latencies (C), and the time spend in each quadrant (abbreviated as Qua) (E) in hidden‐platform test on the fifth day. D) Learning curves show mean daily escape latencies on day 3, 4, and 5. F) There was no difference in the average locomotion velocity of three groups. G) Average number of platform crossings (swims over former platform location). H) 2 days of shuttle box test recording the number of failures in the ten successful shuttles. Records of the time of the three groups staying in the novel arm and exploring new object by I) the Y‐maze test and K) the object recognition test. J) Results from the three groups of the open field test with no different motion speed. (In each graph, the scatters represent the distribution and number of individual mice, and the bar graph shows the mean and error. *n* = 8–11 mice per group; all data shown as mean ± SEM, *P < 0.05, **P < 0.01).

Clinical‐grade hUC‐MSCs were prepared, and quality tests including morphology, phenotype, and differentiation potential were performed (Figure S1, Supporting Information). 2 months after the transplantation of hUC‐MSCs, animals were tested in a series of behavioral experiments including Morris water maze (MWM), Shuttle box, Y‐maze, open field and object recognition for spatial learning and memory ability (**Figure** **1**A). In the MWM task, the escape latency of the SAMP8‐phosphate buffered saline (PBS) (P8‐PBS) group was much longer than that of the normal SAMR1 (R1) mice and MSC treated SAMP8 (P8‐MSC) mice groups on day 3, 4, and 5 (Figure 1D). The typical escape way (Figure 1B,C) and the time spend in each quadrant (Figure 1E) of the R1 and SAMP8‐MSC groups showed immediate and orientated on the fifth day, while locomotion velocity was not significantly different among the three groups (Figure 1F). After the hidden platform was removed, the number of times the mice crossed the original platform location within 1 min was quantified as an index of spatial memory of the platform; the number of crossings of the P8‐PBS group was significantly lower than that of the R1 and P8‐MSC groups (Figure 1G). In 2 days of the shuttle box test, the number of failures in 10 successful shuttles of the R1 and P8‐MSC groups was much lower than that of the P8‐PBS group, indicating that the R1 and P8‐MSC groups had better learning and memory ability in the correlation between light, beeping, and electricity (Figure 1H). In the open field test, the R1 and P8‐MSC groups stayed longer in the central area than did the PBS group, while locomotion velocity was not significantly different among the three groups (Figure 1G). In the Y‐maze test (Figure 1I) and the object recognition test (Figure 1K), the time spent by R1 and P8‐MSC groups in the novel arm and in exploring new objects was longer than that the P8‐PBS group, suggesting that R1 and MSC‐treated AD mice had a stronger memory of the original environment. These data indicate that the transplantation of hUC‐MSCs improves cognitive function in AD mice compared to PBS‐treated AD mice.

### 2.2 hUC‐MSCs Regulated the Expression of AD‐Related Key Proteins and Rejuvenated Endogenic Neurogenesis in AD Mouse Brain

**Figure 2:**
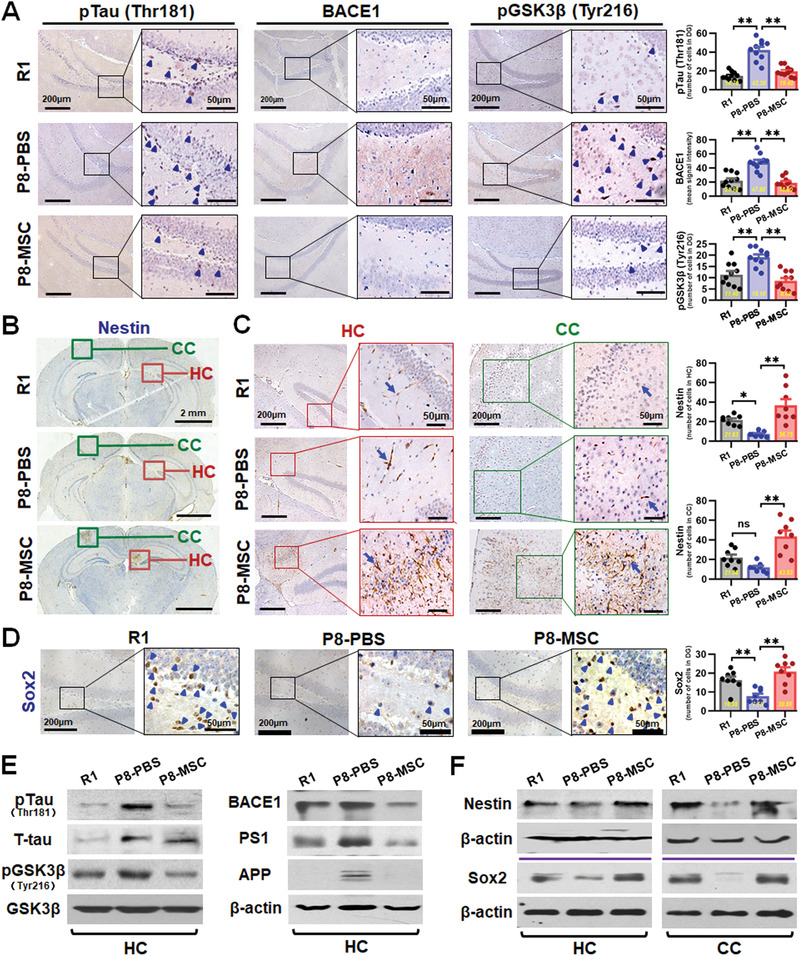
hUC‐MSCs regulated the expression of AD‐related key proteins and improved neurogenic regeneration in SAMP8 mouse brain. A) Representative images of hippocampal dentate gyrus (DG) showing the expressions of pTau (Thr181), BACE1, and pGSK3*β* (Tyr216) in R1, P8‐PBS and P8‐MSC mice by immunohistochemical method. Squares in higher‐magnification inserts indicate the protein positive cells with arrowheads‐labeled individual cell. Right: quantification of pTau (Thr181), BACE1, and pGSK3*β* (Tyr216) in DG region of the R1, P8‐PBS, and P8‐MSC groups. B,C) Immunohistochemical analysis the effects of hUC‐MSCs on the endogenous neurogenesis. B) Representative images illustrated the overall distribution of the Nestin^+^ stem cells from the coronal hippocampal sections. Green circles and red circles respectively represent the specific region of cortex and hippocampus which Nestin^+^ stem cells were concentrated and further showed the higher‐magnification in (C). Images and the number statistics mice for proliferative around CA1 region (C, left) and specific region of cortex (C, right). D) Images and the number statistics for proliferative Sox2^+^ stem cells in DG. E) Western blotting results showing the expressions of pTau (Thr181), pGSK3*β* (Tyr216), APP, BACE1, and PS1 in the hippocampus of the R1, P8‐PBS, and P8‐MSC groups. F) Western blotting results showing the expressions of Nestin and Sox2 in hippocampus and cortex of the three groups. (*n* = 8–10 per group; all data shown as mean ± SEM, *P < 0.05, **P < 0.01).

To determine whether improved cognitive function was accompanied by changes in key proteins in the brain, we measured the levels of several representative proteins within the hippocampus and cerebral cortex of the three groups of mice using immunohistochemical and/or western blotting analysis. The expressions of AD related p‐Tau (Thr181), BACE1, pGSK3*β* (Tyr216), *β*‐amyloid precursor protein (APP) and presenilin 1 (PS1) were significantly decreased in the MSC‐treated group in hippocampus (**Figure** **2**A,E) and/or cerebral cortex (Figure S2, Supporting Information) compared with that in the PBS group. It is clear that a growing body of work supports that impaired hippocampal adult neurogenesis underlies cognitive dysfunction observed in AD.^[^ ^16^ ^]^ Preclinical studies show that the enhancement of endogenous neurogenesis and neuronal plasticity can reverse cognitive impairment.^[^ ^17^, ^18^ ^]^ To explore whether hUC‐MSCs affected endogenous neurogenesis, we analyzed the coronal hippocampal sections of mice for proliferative Nestin^+^ and SOX2^+^ stem cells. The results revealed a decrease in the number of Nestin^+^ stem cells around CA1 region and specific regions of the cortex (Figure 2B,C,F) and SOX2^+^ stem cells in the dentate gyrus (DG) (Figure 2D,F) in the P8‐PBS group, compared with that in the R1 and P8‐MSC groups (Figure 2B–D,F). In addition, we confirmed that exposure of primary mouse hippocampal NSCs to hUC‐MSCs‐CM resulted in increased proliferation (Figure S11, Supporting Information) in vitro. Together, these results demonstrate that hUC‐MSCs regulate the expression of key AD‐related proteins, which could alleviate neuronal damage, and hUC‐MSCs can activate endogenous neurogenesis, which is beneficial for stabilizing hippocampal neural network.

### 2.3 hUC‐MSCs Restored Okadaic Acid‐Induced Neural Cell Damage in an In Vitro AD Cell Model

**Figure 3:**
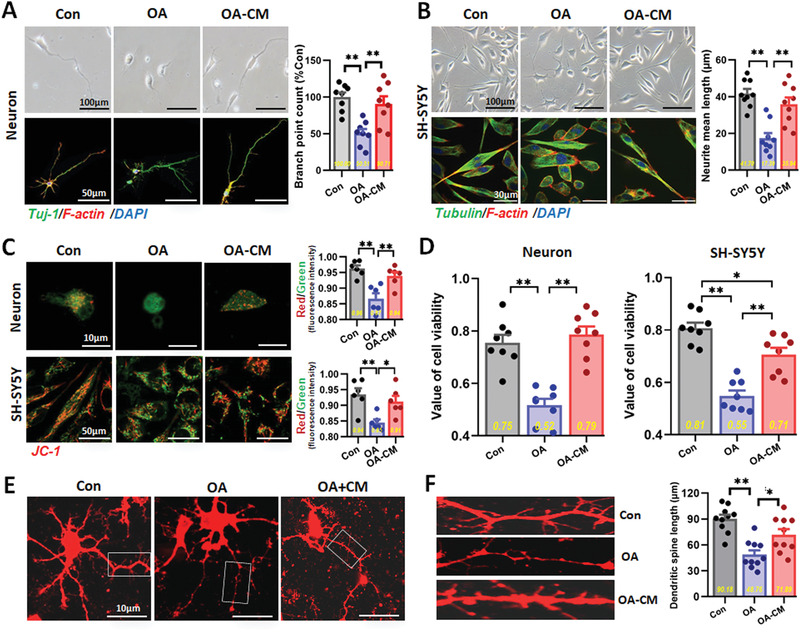
hUC‐MSCs restored OA‐induced neural cell damage in an in vitro AD cell model. A) Representative optical morphological images (upper) and confocal laser scanning images (lower) of the primary neurons, and the quantification of the skeleton branch point by the high‐content cytometer and Thermo Cellomics CellInsight CX5 analysis system was showed on the right (*n* = 8). B) Representative morphological images of SH‐SY5Y cells observed by light microscope (upper) and immunofluorescence method (lower), and the quantification of the skeleton branch point or the neurite mean length by the high content analysis was showed on the right (*n* = 9 per group). C) JC‐1‐labeled the primary neurons and SH‐SY5Y cells for testing the MMP which was observed with confocal microscopy (left) and the quantification of red/green fluorescence intensity by the high‐content cytometer (right) (*n* = 6 per group). D) CCK‐8 assay for the viability of the neurons (left) and SH‐SY5Y cells (right) which had higher viability after CM treating (*n* = 8 per group). E) Confocal laser scanning images of Dil‐filled primary neurons of three groups in culture. Low‐power image of a typical, medium‐sized neuron. Analyses of spine density and morphological parameters were taken from secondary dendrites (box). F) Confocal laser scanning images and the quantification of secondary dendrites (box) from Dil‐filled primary neurons (left), and quantification of the spine length (right) by the high‐content cytometer (*n* = 10 per group). (All data shown as mean ± SEM, *P < 0.05, **P < 0.01).

To further explore whether hUC‐MSCs could restore damaged neural cells, we used an in vitro AD cell model of tauopathy induced by okadaic acid (OA) which we have established in a previous work.^[^ ^19^ ^]^ According to different sensitivities to OA, primary neurons and SH‐SY5Y cells were induced by incubation with OA at a final concentration of 10 nm for 4 h and 20 nm for 24 h (Figure S3, Supporting Information). We analyzed the cell morphology, mitochondrial function, cell viability, subcellular structure and phosphorylation level of tau protein to assess the degree of damage induced by OA and the restorative effects with conditioned medium (CM) of hUC‐MSCs. In terms of cell morphology, primary neurons in the Con group presented polygonal bodies, with longer axons and abundant dendrites; after induced by OA, cell bodies adopted a round morphology, and axons were shortened, bent, and fractured. In contrast, the cell morphology of the CM treatment group was restored and maintained (**Figure** **3**A). The cytoskeleton of primary neurons was labeled with specific fluorescence, and high content analysis revealed that the number of cytoskeleton branches (Figure 3A, right) and total length of neurite protrusion (data not shown) of neurons in the OA group was significantly decreased, while that of neurons in the CM group were recovered. The cell morphology of SH‐SY5Y cells showed similar characteristics to those of primary neurons (Figure 3B). We subsequently used JC‐1 to label primary neurons and SH‐SY5Y cells for testing the mitochondrial membrane potential (MMP) which reflects the early apoptosis of cells. High content analysis revealed that the MMP of neurons and SH‐SY5Y cells were significantly reduced in the OA group, while the MMP in the CM‐treated group achieved partial recovery (Figure 3C). Both neurons and SH‐SY5Y cells had higher viability after CM treatment (Figure 3D). To verify whether hUC‐MSCs could influence the size of dendritic spines which constitute the main locus of excitatory synaptic interaction among central neurons, Dil dye staining was performed on the three groups (Figure 3E,F). Dil‐filled primary neurons had an integral shape as observed with confocal laser scanning. Analyses of spine parameters were obtained from secondary dendrites (box) (Figure 3E). The length and density of secondary dendrites from Dil‐filled primary neurons in the OA group were less than those in the Con group, but the size of dendritic spines was distinctly restored after hUC‐MSCs treatment (Figure 3F). Based on neuronal tau hyperphosphorylation in AD cell models, we tested several susceptive phosphorylation sites of tau protein in OA‐treated SH‐SY5Y cells using western blotting and observed that hUC‐MSCs partly inhibited tau hyperphosphorylation (Figure S4, Supporting Information). These results indicate that hUC‐MSCs effectively recover and rescue OA‐induced damage to neural cells in an in vitro AD cell model.

### 2.4 HGF Secreted from hUC‐MSCs Rescued the AD Cell Model from OA‐Induced Neural Cell Damage

**Figure 4:**
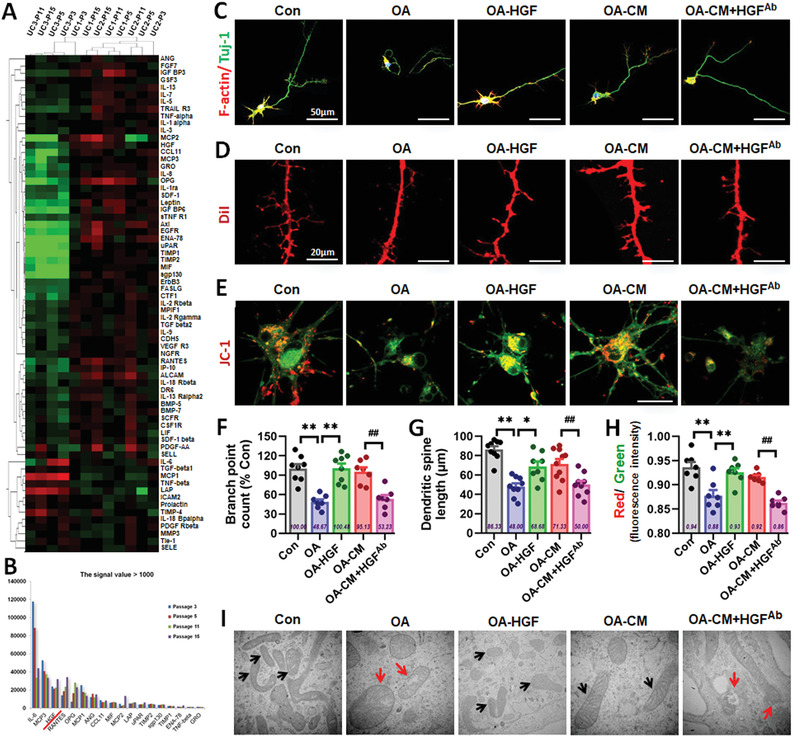
HGF secreted from hUC‐MSCs rescued the AD cell model from OA‐induced neural cell damage in vitro. A) The heat map of the cytokines in hUC‐MSCs‐CM of four different generations (passage 3, 5, 11, 15) from three different umbilical cords (UC1, 2, 3)‐derived hUC‐MSCs. B) 18 factors expressing the strongest signal with the value greater than 1000. C) Confocal laser scanning images of F‐actin (red)/Tuj‐1 (green) labeled neurons in Con, OA, OA‐HGF, OA‐CM, and OA‐CM+HGF‐Ab groups. D) Confocal laser scanning images of Dil‐filled primary neurons of the five groups in culture. E) Representative images of JC‐1‐labeled neurons of the five groups. F–H) The quantification of the skeleton branch point (F), dendritic spine length (G), and red/green fluorescence intensity (H) by the high‐content cytometer Thermo Cellomics Cell Insight CX5 analysis system. I) Morphometric ultrastructural analyses from transmission electron microscope (TEM) showing the intracellular mitochondria of SH‐SY5Y in the five groups. (*n* = 7–10 per group; all data shown as mean ± SEM, *P < 0.05, **P < 0.01; ^##^ *P* < 0.01).

After clarifying the effects of hUC‐MSCs in AD models in vivo and in vitro, we focused on the mechanism by which core functional factors secreted from hUC‐MSCs played important roles in hUC‐MSC‐modulated recovery of cognitive function and neurological damage. We used high throughput screening (HTS) to detect the quantity of expression of 174 known cytokines in the hUC‐MSC‐CM of four different generations (passage 3, 5, 11, 15) from three different umbilical cords (UC1, 2, 3)‐derived hUC‐MSCs. We obtained the cytokine expression spectrum of 12 samples and analyzed the overall quality control of the data. We found stable and reliable data through cluster analysis. From the results of the heat map (**Figure** **4**A), factors from hUC‐MSCs of UC1 and UC2 shared a higher similarity than that of UC3, which was identical to the proliferation ability among the three sources of hUC‐MSCs (data not shown). We determined 18 factors that expressed the strongest signal with values greater than 1000 (Figure 4B). After investigating and analyzing the role of each factor in detail, we highlighted four factors including IL‐6, HGF, ANG, and GRO, which may be involved in repairing damage. Using the OA‐induced damage model in vitro, we observed that only HGF could dramatically restore the damaged primary neurons (Figure S5A,B,E,F, Supporting Information) and SH‐SY5Y cells (Figure S5C,D, Supporting Information) to approximate the state of the Con groups. IL‐6 exhibited a degree of reparative ability, whereas ANG and GRO had no reparative effects in the model.

We then focused on the function of HGF and explored whether HGF secreted from hUC‐MSCs could mediate hUC‐MSC‐modulated recovery of neurological damage. We examined the cytoskeleton, dendritic spines, and mitochondrial function (Figure 4C–E) in OA‐induced primary neurons in vitro with laser confocal microscopy and analyzed the cytoskeleton branches, dendritic spine length, and MMP using high‐content analysis (Figure 4F–H). The number of cytoskeleton branches of the neurons was significantly recovered in the HGF‐treated group to that of the Con and MSC‐treated groups (Figure 4C,F). Analyses of dendritic spine length revealed that the size of dendritic spines in the HGF group was similar to that of the CM groups (Figure 4D,G). The MMP in the HGF group exhibited evident recovery to the level of the CM group (Figure 4E,H). These results suggested that HGF effectively recovered and rescued OA‐induced damage to neurons in the AD cell model in vitro. To explore whether HGF secreted from hUC‐MSCs could mediate hUC‐MSC‐modulated recovery of neurological damage, a neutralizing antibody against HGF (HGF^Ab^) was added into the MSC‐treated group. The repair capabilities of hUC‐MSCs were dramatically restrained compared with that of the CM group (Figure 4C–H). We also used the SH‐SY5Y cell model of OA‐induced damage and investigated whether HGF^Ab^ weakened the capacity of CM to recover neurite length and proliferation ability (Figure S6, Supporting Information). Morphometric ultrastructural analyses with a transmission electron microscope indicated that the intracellular mitochondria of OA‐damaged SH‐SY5Y cells displayed collapse, swelling, and vacuoles with a large number of secondary lysosomes and residues. After treatment with HGF and CM, the intracellular mitochondria of treated SH‐SY5Y cells restored their shape to long ellipse, and mitochondrial cristae were arranged neatly. In contrast, the reparative effects of CM were dramatically restrained after the addition of HGF^Ab^ (Figure 4I). Taken together, these data support an important role for HGF secreted from hUC‐MSCs in mediating hUC‐MSC‐modulated recovery of neurological damage in vitro.

### 2.5 HGF Improved Cognitive Function and Regulated the Expression of Key AD‐Related Proteins in SAMP8 Mice

**Figure 5:**
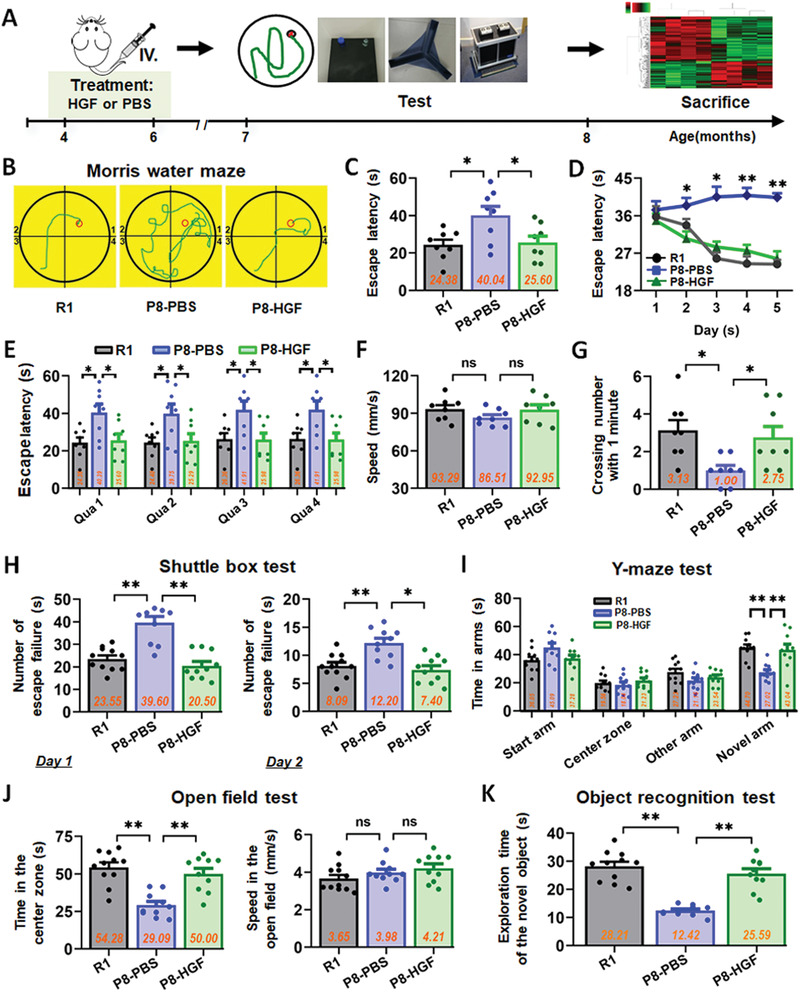
HGF improved spatial learning and memory ability in SAMP8 mice. A) Schematic illustrating the chronological order used for HGF or PBS treatment, MWM, shuttle box, Y‐maze, open field and object recognition testing. B–G) Results from R1, P8‐PBS, and P8‐HGF groups that were cognitively tested by MWM. The typical escape way (B), the escape latencies (C), and the time spend in each quadrant (E) in hidden‐platform test on the fifth day. D) Learning curves show mean daily escape latencies on day 2, 3, 4, and 5. F) There was no difference in the average locomotion velocity of three groups. G) Average number of platform crossings (swims over former platform location). H) 2 days of shuttle box test recording the number of failures in the ten successful shuttles. Records of the time of the three groups staying in the Novel arm and exploring new object by I) the Y‐maze test and K) the object recognition test. J) Results from the three groups of the open field test with no different motion speed. (*n* = 8–11 mice per group; all data shown as mean ± SEM, *P < 0.05, **P < 0.01).

To further explore whether HGF could improve hippocampal function and enhance cognitive ability in vivo, we administered the SAMP8 mice with 8 weeks of HGF treatment and tested animals in a series of behavioral experiments MWM, open field and object recognition, Y‐maze, and Suttle box to assess spatial learning and memory ability (**Figure** **5**A). In the MWM task, the escape latency of the P8‐PBS group was longer than that of the R1 and P8‐HGF groups on day 2, 3, 4, and 5 (Figure 5D). The typical escape way (Figure 5B,C) and the time spend in each quadrant (Figure 5E) of the R1 and P8‐MSC groups showed immediate and orientated on the fifth day, while locomotion velocity was not significantly different among the three groups (Figure 5F). After the hidden platform was removed, the number of times mice crossed the original platform location within 1 min was quantified as an index of spatial memory of the platform. The number of crossing times of the P8‐PBS group was significantly lower than that of the R1 and P8‐HGF groups (Figure 5G). In the shuttle box test, R1 and HGF‐treated AD mice completed the vested shuttles faster than did the PBS group, suggesting that the HGF group had better learning and memory ability in the correlation between light, sound, and electricity (Figure 5H). In the open field test, the R1 and HGF groups stayed in the central area longer than did the PBS group, while locomotion velocity was not significantly different between the two groups (Figure 5J). In the Y‐maze test (Figure 5I) and object recognition test (Figure 5K), the time spent in the novel arm and in exploring new objects was longer in the R1 an HGF groups than in the PBS group, suggesting that HGF‐treated AD mice had stronger memories of the original environment. To further explore the function of HGF on hippocampal synaptic plasticity in SAMP8 mice, we tested hippocampal long‐term potentiation which is thought to underpin learning and memory. The HGF group exhibited a higher slope of the field excitatory postsynaptic potentials than those of the PBS group, suggesting that HGF could enhance hippocampal synaptic plasticity in SAMP8 mice (Figure S7A, Supporting Information). Moreover, we tested fear conditioning responses of SAMP8 mice and the results indicated that HGF‐treated mice had stronger associative memory of sound and electricity (Figure S7B, Supporting Information).

The neuropathological hallmarks of AD and other tauopathies include senile plaques, neurofibrillary tangles, and/or neuroinflammation; these are evident in SAMP8 mice. SAMP8 brains over‐produce APP, have increased tau phosphorylation, accompanied with gliosis and neuroinflammation.^[^ ^20^, ^21^, ^22^ ^]^ Indeed, previous studies have reported on tau phosphorylation dynamics in SAMP8 mice, and various forms of hyperphosphorylated tau are more highly expressed in SAMP8 mice than in SAMR1 mice.^[^ ^23^, ^24^, ^25^ ^]^ We visualized the NFT‐like pathology in hippocampal neurons in SAMP8 mouse brains by thioflavin‐S staining and found that PBS‐treated mice had more NFTs in the DG region than that in SAMR1 mice. Both MSC and HGF‐treated groups had lower number of NFTs in the DG region than that of the PBS group, and A*β* distribution was similar to NFT distribution (Figure S8, Supporting Information). These effects were partly associated with reversal of disease‐associated microglial neuroinflammation, as evidenced by decreased microglia‐induced proinflammatory cytokines (IL‐1*β* and TNF‐*α*) and increased anti‐inflammatory cytokine IL‐4, suggesting that both hUC‐MSCs and HGF produced sustained neuroprotective effects (Figure S9, Supporting Information). Taken together, these data indicate that HGF improves cognitive function and regulates the expression of key AD‐related proteins in SAMP8 mice.

### 2.6 Inhibition of HGF Secretion Weakened the Capacity of hUC‐MSCs to Enhance Spatial Learning and Memory Ability in SAMP8 Mice

**Figure 6:**
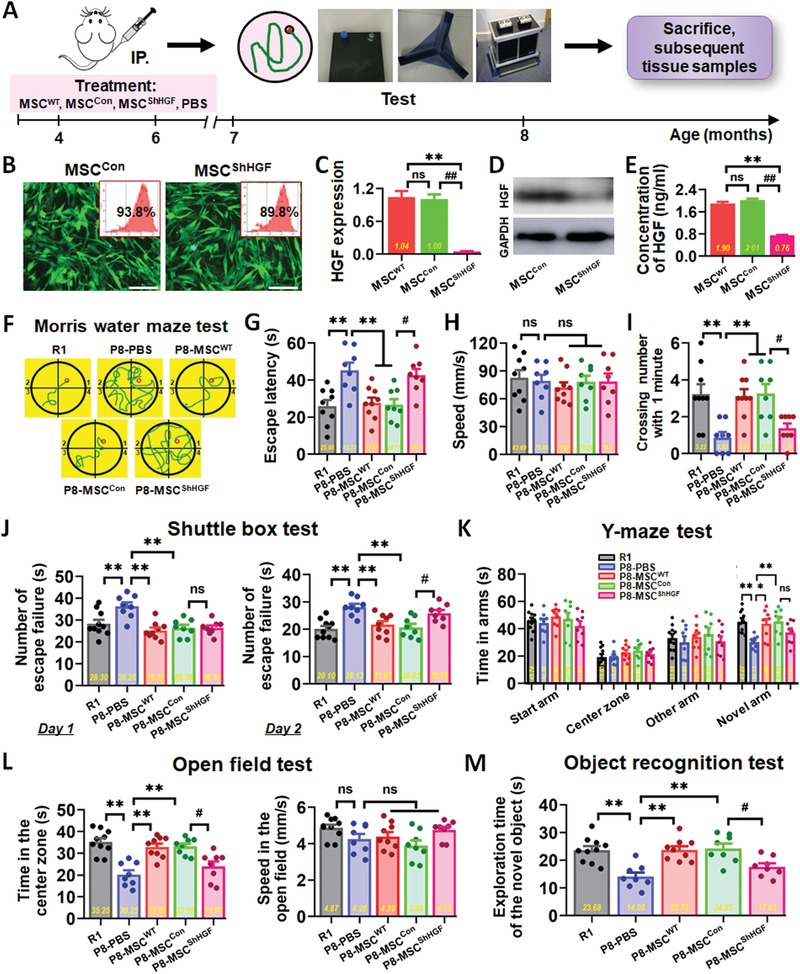
Inhibition of HGF signaling weakened the capacity of hUC‐MSCs to enhance spatial learning and memory ability in SAMP8 mice. B) HGF‐deficient hUC‐MSCs (MSC^ShHGF^) and control cells (MSC^Con^) were constructed, and wild‐type hUC‐MSCs (MSC^WT^) were used as positive control. The GFP positive rate reached about 90%, indicating a high transfection efficiency. The expression level of HGF in MSC^ShHGF^ and MSC^Con^ was tested using C) quantitative real‐time PCR (qPCR), D) Western blotting, and E) ELISA methods, and the expression level of HGF in MSC^ShHGF^ was much lower than that in MSC^Con^ and MSC^WT^ (n = 3 per group). A) Schematic illustrating the chronological order used for MSC^WT^, MSC^Con^, MSC^ShHGF^, and PBS treatment, MWM, Shuttle box, Y‐maze, open field and object recognition testing. F–I) Results from R1, MSC^ShHGF^, MSC^Con^, and MSC^WT^ groups that were cognitively tested by MWM. The typical escape way (F) and the escape latencies (G) in hidden‐platform test on the fifth day. H) The average locomotion velocity of five groups. I) Average number of platform crossings (swims over former platform location). J) 2 days of shuttle box test recording the number of failures in the ten successful shuttles. Records of the time of the five groups staying in the novel arm and exploring new object by the K) Y‐maze test and M) the object recognition test. L) Results from the five groups of the open field test with no different motion speed. (*n* = 8–10 mice per group; all data shown as mean ± SEM, *P < 0.05, **P < 0.01; ^#^ *P* < 0.05, ^##^ *P* < 0.01).

To confirm whether HGF was the core molecule responsible for recovering the cognitive effects of hUC‐MSCs in vivo, we used HGF‐deficient hUC‐MSCs to verify the critical roles of HGF in SAMP8 mice. First, we constructed HGF‐deficient hUC‐MSCs (MSC^ShHGF^) and control cells (MSC^Con^) (**Figure** **6**B). The expression level of HGF in MSC^ShHGF^ was much lower than that in MSC^Con^ and wild‐type hUC‐MSCs (MSC^WT^) (Figure 6C–E). Then, we administrated SAMP8 mice with 8 weeks of treatment of MSC^WT^, MSC^Con^, MSC^ShHGF^, and PBS (Figure 6A). Subsequently, the animals were tested in a series of behavioral experiments (Figure 6F–M). In the MWM task, escape latency of the P8‐MSC^ShHGF^ group was longer than that of the R1, MSC^WT^, and MSC^Con^ groups on the fifth day (Figure 6F–H). After the hidden platform was removed, the number of times mice crossed the original platform location within 1 min was quantified as an index of spatial memory of the platform. The number of times the P8‐MSC^ShHGF^ group crossed the original platform location within 1 min was significantly lower than that of the P8‐MSC^Con^ group (Figure 6I). In the shuttle box test, MSC^ShHGF^‐treated AD mice completed the vested shuttles more slowly than did R1, MSC^WT^, and MSC^Con^ groups in the second day, implying that the P8‐MSC^ShHGF^ group had poorer learning and memory ability (Figure 6J). In the open field test, the R1, P8‐MSC^WT^, and P8‐MSC^Con^ groups stayed longer in the central area than did the PBS group, but there was no significant difference between the P8‐MSC^ShHGF^ and P8‐MSC^Con^ groups (Figure 6L). In the Y‐maze test (Figure 6K) and object recognition test (Figure 6M), the time spent in the novel arm and exploring new objects was shorter in the MSC^ShHGF^ group than the R1, MSC^WT^, and MSC^Con^ groups, suggesting that MSC^ShHGF^ treated AD mice had poorer memories of the original environment. Taken together, these data from different behavioral experiments indicate that transplantation of hUC‐MSCs improves cognitive function in AD mice, and inhibition of HGF secretion weakens the capacity of hUC‐MSCs to enhance spatial learning and memory ability in SAMP8 mice. This suggests that HGF plays an important role in mediating hUC‐MSC‐modulated recovery of cognitive deficits in SAMP8 mice.

### 2.7 HGF Mediated hUC‐MSCs Regulating tau Hyperphosphorylation via Activation of cMet‐AKT‐GSK3*β* Signaling Pathway

**Figure 7:**
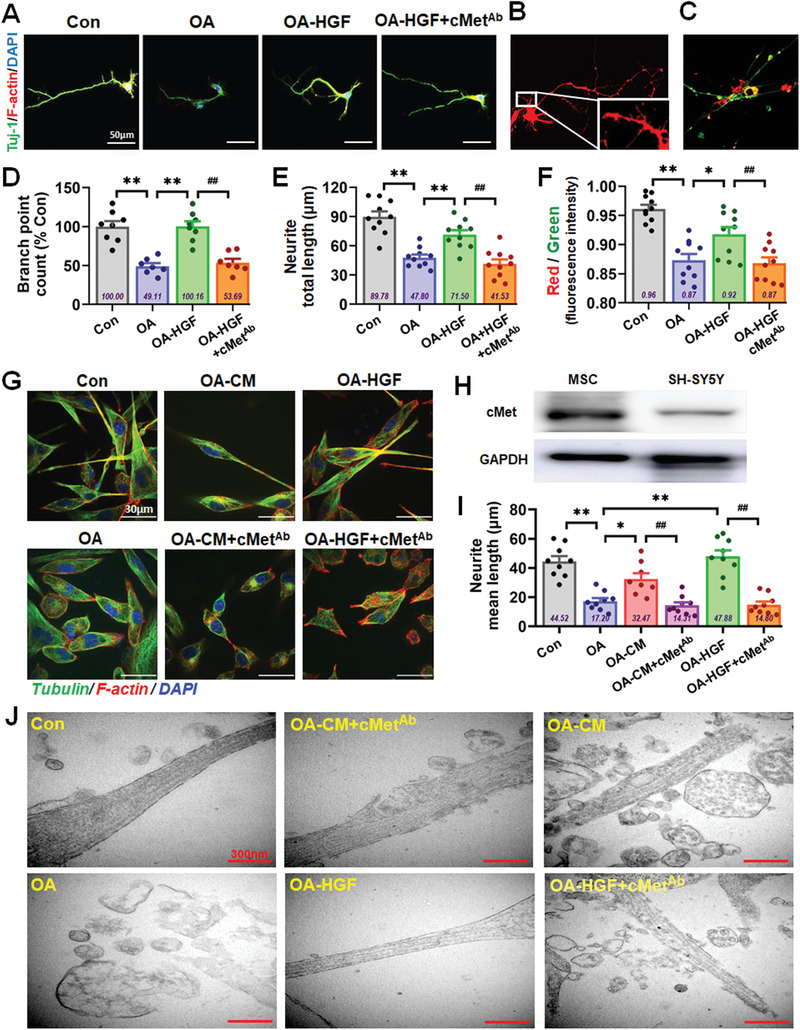
c‐Met played important roles during recovery of damaged neural cells by HGF. A) Confocal laser scanning images of F‐actin (red)/Tuj‐1 (green) labeled neurons in Con, OA, OA‐HGF, and OA‐HGF+cMet‐Ab groups. B) Confocal laser scanning images of Dil‐filled primary neurons of the five groups in culture. C) Representative images of JC‐1‐labeled neurons of the five groups. D–F) The quantification of the skeleton branch point (D), dendritic spine length (E), and red/green fluorescence intensity (F) by the high‐content cytometer and Thermo Cellomics CellInsight CX5 analysis system. G) Confocal laser scanning images of F‐actin (red)/Tubulin (green) labeled SH‐SY5Y cells in Con, OA, OA‐CM, OA‐HGF, OA‐HGF+cMet‐Ab, and OA‐HGF+cMet‐Ab groups. H) The expression level of c‐Met in MSC and SH‐SY5Y cells was tested by Western blotting. I) The quantification of the neurite mean length of the SH‐SY5Y cells in the six groups. J) Morphometric ultrastructural analyses from transmission electron microscope (TEM) showing the cytoskeleton of the neurons in the six groups. (All data shown as mean ± SEM, *P < 0.05, **P < 0.01; ^##^ *P* < 0.01).

We further explored the mechanisms by which HGF mediated hUC‐MSCs regulating restoration of neural damage, structure, and function. First, we directly examined the high affinity HGF receptor, c‐Met, which is able to mediate all the known effects of HGF.^[^ ^26^ ^]^ We used the HGF R/c‐Met antibody (cMet^Ab^) that is able to block more than 90% of binding in OA‐induced primary neurons and SH‐SY5Y cells in vitro (**Figure** **7** and Figure S10, Supporting Information). The cytoskeleton branch number (Figure 7A), dendritic spine length (Figure 7B) and MMP level (Figure 7C) in the HGF group exhibited recovery, but the reparative capabilities of HGF were dramatically restrained when cMet^Ab^ was added (Figure 7D–F). SH‐SY5Y cells (Figure 7G–I; and Figure S10, Supporting Information) showed similar characteristics, whereby the repair effects of CM and HGF were dramatically restrained after the addition of cMet^Ab^. Furthermore, morphometric ultrastructural analyses indicated that the intracellular mitochondria of OA‐damaged neurons displayed collapse, swelling and disintegration; after treatment with HGF and CM, the cytoskeleton of treated neurons recovered substantially, while the reparative effects of CM and HGF were dramatically restrained after the addition of cMet^Ab^ (Figure 7J). These data support c‐Met as an important receptor for HGF that mediates the function of HGF for restoring neurological damage in vitro.

**Figure 8:**
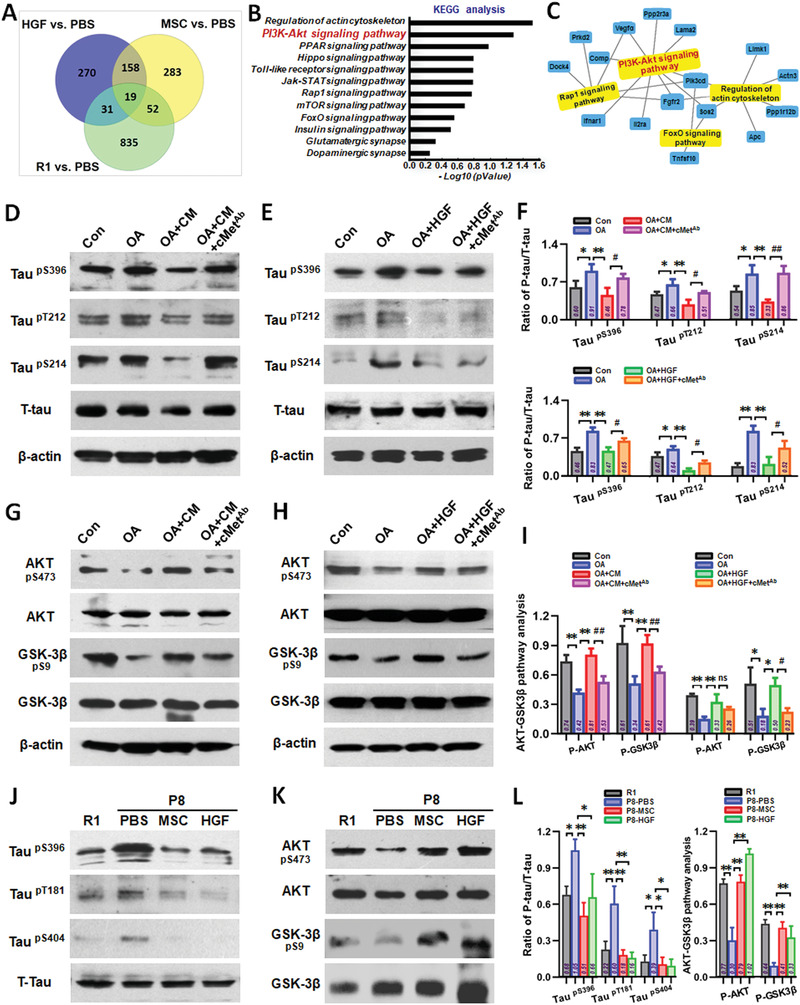
The HGF‐cMet‐AKT‐GSK3*β* axis regulated the hyperphosphorylation of protein tau. A) Differentially expressed genes were identified using the raw data from the 16 hippocampal tissue samples. 19 different genes were obtained after a 1.5‐FC with respect to PBS group in gene expression. B) KEGG pathway analysis evaluated the enriched pathways for the representative profiles of genes involved in signal transduction pathways. C) Pathway analysis represented more genes related with PI3K‐Akt signaling pathway. D) Representative Western blotting results showing the expression of phospho‐Tau (Ser396, Thr212, and Ser214) of SH‐SY5Y cells in Con, OA, OA‐CM, and OA‐CM+cMet‐Ab groups. E) Representative Western blotting results showing the expression of phospho‐Tau (Ser 396, Thr212, and Thr214) of SH‐SY5Y cells in Con, OA, OA‐HGF, and OA‐HGF+cMet‐Ab groups. F) The quantification of phospho‐Tau (Ser396, Thr212, and Ser214) compared with total‐Tau for (D) and (E). G) Representative Western blotting results showing the expression of phospho‐AKT (Ser473) and phospho‐GSK3*β* (Ser9) of SH‐SY5Y cells in Con, OA, OA‐CM, and OA‐CM+cMet‐Ab groups. H) Representative Western blotting results showing the expression of phospho‐AKT (Ser473) and phospho‐GSK3*β* (Ser9) of SH‐SY5Y cells in Con, OA, OA‐HGF, and OA‐HGF+cMet‐Ab groups. I) The quantification of phospho‐AKT (Ser473) and phospho‐GSK3*β* (Ser9) respectively compared with total‐AKT and total‐GSK3*β* for (G) and (H). J) Phosphorylation of tau (Ser396, Thr181, and Ser404) and K) phosphorylation of AKT (Ser473), GSK3*β* (Ser9) in R1, P8‐PBS, P8‐MSC, and P8‐HGF mice. L) The quantification of phospho‐Tau (Ser396, Thr181, and Thr404) compared with total‐Tau (left), and the quantification of phospho‐AKT (Ser473) and phospho‐GSK3*β* (Ser9) respectively compared with total‐AKT and total‐GSK3*β* (right). (*n* = 3–4 per group; all data shown as mean ± SD, *P < 0.05, **P < 0.01; ^#^ *P* < 0.05, ^##^ *P* < 0.01).

Second, we used an exploratory hippocampal tissue‐based mRNA microarray to analyze and search for candidate pathways. We divided hippocampal tissue samples into four groups: R1 mice, and three different treatments (PBS, hUC‐MSC, and HGF) in SAMP8 mice. Every group had four different hippocampal tissue samples. After obtaining the raw data from 16 hippocampal tissue samples, differentially expressed genes were identified. When a 1.5‐FC with respect to gene expression of the PBS group was used to filter our gene list, 19 different genes were obtained, which we subsequently focused on (**Figure** **8**A). With the aim of exploring the relationship between differentially expressed genes and probable pathways, KEGG pathway analysis was used to evaluate the pathways enriched for the representative profiles of genes involved in signal transduction pathways (Figure 8B), such as Regulation of actin cytoskeleton, PI3K‐AKT signaling pathway, PPAR signaling pathway, Rap1 signaling pathway, FoxO signaling pathway. The activation of AKT, which inhibits GSK3*β*, is one of the most well characterized cell survival signaling pathways.^[^ ^27^ ^]^ Pathway analysis certified that more genes were related with PI3K‐AKT signaling pathway (Figure 8C). We examined the immunoblot of p‐AKT (Ser473) and p‐GSK3*β* (Ser9) in OA‐treated SH‐SY5Y cell model (Figure 8G,H). Immunoblot of p‐AKT and p‐GSK3*β* was decreased in OA‐treated SH‐SY5Y cells compared to that of the control group. Treatments with CM and HGF overcame this effect and significantly increased the immunoblot reactivity of p‐AKT and p‐GSK3*β* in OA‐treated cells compared to that of OA‐treated cells alone; while, the positive effects of phosphorylation of AKT and GSK3*β* by CM and HGF were partly restricted after the addition of cMet‐Ab (Figure 8G–I).

To investigate whether HGF could mediate hUC‐MSCs and regulate the phosphorylation of tau protein, we analyzed the phosphorylation of tau at Ser396, Thr212, and Ser214 by immunoblot in an OA‐treated SH‐SY5Y cell model (Figure 8D,E). OA treatment significantly increased the phosphorylation of tau at Ser396, Thr212, and Ser214 compared to that of control. Both CM and HGF treatments significantly lowered OA‐induced tau phosphorylation at these three sites compared to OA treatment alone. The suppression effects of tau phosphorylation by CM and HGF were partly restricted after the addition of cMet‐Ab (Figure 8D–F). We also investigated the level of tau phosphorylation and AKT/GSK3*β* in the hippocampus of P8 mice (Figure 8J–L). Phosphorylation of tau at Ser396, Thr181, and Ser404 was significantly increased in PBS‐treated P8 mice compared to that in R1 mice. Both hUC‐MSCs and HGF treatments significantly lowered tau phosphorylation at the three sites in P8 mice compared to that in the PBS group (Figure 8J,L‐left). Phosphorylation of AKT (Ser473) and GSK3*β* (Ser9) was decreased in PBS‐treated P8 mice compared to that in R1 mice, and treatments with hUC‐MSCs and HGF restored the level of p‐AKT and p‐GSK3*β* in P8 mice compared to that in the PBS‐treated group (Figure 8K,L‐right).

## 3 Discussion

According to the WHO's latest top 10 global causes of death, deaths due to AD and other dementias more than doubled between 2000 and 2016, making it the fifth leading cause of global deaths in 2016 compared to 14th in 2000 (http://www.who.int/news-room/fact-sheets/detail/the-top-10-causes-of-death). AD exerts a physical, psychological, social, and economical effects, on people with AD as well as their carers, families and society at large. Currently, there are no treatments to cure AD or alter its course of progression. As such, novel AD treatments are a critical unmet need.

Recently, the transplantation of stem cells has been shown to be effective treating neurodegenerative diseases.^[^ ^7^, ^8^ ^]^ Numerous animal studies have shown positive effects of stem cells (including BMSCs, ADMSCs, or NSCs) in AD treatment, which could improve cognitive performance via diverse immunological, histological, and genetic measures.^[^ ^9^, ^10^, ^11^, ^12^, ^13^ ^]^ Nevertheless, these stem cells have many factors that limit their clinical application, including low yield, ethical issues, and invasive procedures. hUC‐MSCs have higher yield, and invasive procedures without ethical issues. Importantly, hUC‐MSCs maintain an earlier embryologic phase, are much younger, and can secrete a wide range of multifunctional factors. These features indicate that hUC‐MSCs may be a better choice for clinical application than many other MSCs.

However, the use of hUC‐MSCs derived from different sources and using various methods further confounds the direct comparison of findings from different labs. For clinical application, a standardized protocol that guarantees the reproducibility, safety, and efficacy of hUC‐MSCs is of utmost importance.^[^ ^28^ ^]^ Indeed, while the use of animal components carries the risk of zoonoses and xenogeneic immune reactions,^[^ ^29^ ^]^ laboratory‐generated hUC‐MSCs do not undergo quality and safety testing that is required for clinical applications. Our group has established a Cell Factory and Biobank at the South China Institute of Biomedicine in Guangzhou that is currently producing clinical‐grade hUC‐MSCs, using methods^[^ ^14^ ^]^ that are fully compliant with current Good Manufacturing Practice guidelines, and conform to the quality standards of National Institutes for Food and Drug Control.

Our past work has characterized beneficial effects of clinical‐grade hUC‐MSC‐based therapies for intervening with normal aging‐related cognitive dysfunction.^[^ ^14^ ^]^ However, whether hUC‐MSCs have effects on pathological cognitive decline in AD, needs to be further addressed. Although some studies have showed positive effects of hUC‐MSCs in the treatment of AD,^[^ ^30^, ^31^ ^]^ little is known about which core factor secreted from hUC‐MSCs plays important roles in hUC‐MSC‐modulated recovery of cognition function, which may be one of the key factors restricting the better clinical application of hUC‐MSCs. Therefore, it is very essential to clarify how hUC‐MSCs play a therapeutic role in AD from the perspective of hUC‐MSCs themselves, which may be the crux of both basic research and clinical application of hUC‐MSCs in the treatment of AD. Here, we comprehensively evaluated for the first time the effects of clinical‐grade hUC‐MSCs on AD and explored whether clinical‐grade hUC‐MSCs could improve cognitive ability in SAMP8 mice. We further demonstrated the core functional factor HGF secreted from hUC‐MSCs plays important roles in hUC‐MSC‐modulated recovery of damaged neural cells at least partially through the activation of cMet‐AKT‐GSK3*β* signaling pathway.

Selecting and confirming better experimental models was key part of this study. AD is predominantly a sporadic late‐onset disease with exponentially increasing prevalence from the age of 65 years old.^[^ ^32^ ^]^ The majority of cases are most likely caused by complex interactions among multiple genetic, epigenetic, and environmental factors. Therefore, the spontaneous SAMP8 strain has several distinct advantages over gene‐modified models and is particularly well suited to study the “transitional switch” between aging and AD. The phenotypes of the SAMP8 mouse resemble the symptoms of late‐onset and age‐related sporadic AD patients.^[^ ^33^, ^34^ ^]^ We therefore used SAMP8 mice as an animal model to study AD. SAMP8 mice that received clinical‐grade hUC‐MSC grafting exhibited improved performance, as assessed by five well characterized and widely used cognitive tasks (Figure 1). These indicative or spontaneous exploration tasks interrogate various combinations of hippocampal and cortical learning and memory processes, providing a quantifiable readout of behavioral performance between cohorts. We then defined the therapeutic function of clinical‐grade hUC‐MSCs on SAMP8 mice at histological and protein levels, including regulation of representative AD‐related key proteins p‐Tau (Thr181), BACE1, pGSK3*β* (Tyr216), APP, PS1, pGSK3*β* (Ser9), and pAKT (Ser473) (Figures 2 and 8K; Figure S2, Supporting Information); A*β* plaques, NFTs and neuroinflammation in the hippocampus (Figures S8 and S9, Supporting Information); and the regeneration of endogenous neural stem cells (Figure 2). Our results above indicated that clinical‐grade hUC‐MSCs have a significant effect on the intervention of AD pathological indicators based on A*β* and tau, and have great potential for the regeneration and repair of damaged neural cells. Although the multiple regulatory links involved still need to be clarified gradually, this lays an important theoretical foundation to advance preclinical and clinical studies of hUC‐MSCs.

Selecting the AD cell model in vitro is also important to further explore the function and mechanisms by which hUC‐MSCs restores damaged neural cells. Based on the two major pathological characteristics of AD patients, a common AD cell model in vitro is to increase A*β* levels or induce tauopathy in cultured neural cells. As a result of the amyloid cascade hypothesis, efforts to develop therapies for AD have focused mainly on reducing levels of A*β* in the brain,^[^ ^35^ ^]^ but more than 100 candidate treatment compounds have failed in this attempt.^[^ ^36^, ^37^ ^]^ While growing research has indicated that hyperphosphorylated tau and NFTs seem more strongly correlated with cognition in multivariate analyses and synaptic and neuronal loss, which are closely associated with memory deficits. Indeed, more new light has been shed on the possible interactions of A*β* and tau, and novel findings have shifted our understanding of the role of tau which acts downstream of A*β* or toward being a crucial partner of A*β* to induce neuronal death in the pathogenesis of AD,^[^ ^5^, ^6^ ^]^ which prompts that targeting tau pathology seems more clinically effective than single A*β*‐directed therapies.^[^ ^37^, ^38^ ^]^ Here, we used an in vitro AD cell model of tauopathy induced by OA which is a classic method to study tau diseases in vitro^[^ ^39^, ^40^ ^]^ that we have established in previous work.^[^ ^19^ ^]^ By using this model, we demonstrated that hUC‐MSCs could effectively recover and rescue OA‐induced neurological damage, including improving cytoskeleton arrangement and mitochondrial function, restoring dendritic length and dendritic spines number, and partially inhibiting tau hyperphosphorylation (Figure 3 and Figure S4, Supporting Information). Through in vivo and in vitro models, we have more comprehensive evidence that hUC‐MSCs could effectively treat AD, at least partially by regulating tau hyperphosphorylation.

Once thought to function in cell replacement for damaged tissue‐resident cells, it is now widely established that the more immediate principal mechanism of action of MSCs in vivo is paracrine in nature, and that the generation of multifunctional factors and extracellular vesicles by MSCs is a critical parameter in their ability to modify the function of host cells and tissues.^[^ ^41^ ^]^ Here, we confirmed that paracrine activity of soluble factors secreted by hUC‐MSCs could effectively recover and rescue neurological damage consistent with our previous studies.^[^ ^14^ ^]^ However, it is unclear which core functional factors secreted from hUC‐MSCs play important roles in hUC‐MSC‐modulated recovery of cognitive function and neurological damages. Here, we detected the supernatant of hUC‐MSCs by HTS and obtained the cytokine expression spectrum of hUC‐MSCs (Figure 4A). We focused on secretory factors expressing the strongest signal (Figure 4B), and combined this with bioinformatics analysis and literature search to analyze the role of each factor in detail (data not shown); we then selected four potential factors including IL‐6, HGF, ANG, and sgp130, which may be involved in damage repair.^[^ ^42^, ^43^, ^44^, ^45^, ^46^ ^]^ With the mature and stable OA‐induced damage model in vitro, several key indexes were investigated to analyze and compare the repair features of the four factors. We observed that only HGF substantially ameliorated neural cell damage (Figure S5, Supporting Information) in multiple ways, including dendritic length and mitochondrial function. IL‐6 had some reparative ability, but ANG and GRO had no repair effects in the model. Therefore, we focused on HGF in subsequent studies. In order to verify whether HGF secreted from hUC‐MSCs could mediate hUC‐MSC‐modulated recovery of neurological damage, a neutralizing antibody against HGF (HGF‐Ab) was added into the MSC‐treated group. We observed that multiple repair capabilities (cytoskeleton, dendritic spines, and mitochondrial function) of hUC‐MSCs were dramatically restrained compared with that in the CM group (Figure 4C–I and Figure S6, Supporting Information). These data supported an important role for HGF secreted from hUC‐MSCs in mediating hUC‐MSC‐modulated recovery of neurological damages in vitro. Further, we demonstrated that HGF alone improved cognitive function (Figure 5 and Figure S7B, Supporting Information), enhanced hippocampal synaptic plasticity (Figure S7A, Supporting Information), and regulated the expression of AD‐related key proteins in SAMP8 mice (Figure 8J–L and Figure S8, Supporting Information). For example, after hUC‐MSCs or HGF injection, the central area activity of SAMP8 mice was increased compared with that in the PBS group in the open field test, suggesting that hUC‐MSCs or HGF treatment alone could improve general locomotor activity of AD mice in novel environments (Figures 1J and 5J). To confirm whether HGF was the core molecule underpinning the in vivo effects of hUC‐MSCs, we used HGF‐deficient hUC‐MSCs to verify the critical roles of HGF in SAMP8 mice. The overall performance in different behavioral experiments indicated that inhibition of HGF secretion could to some extent weaken the capacity of hUC‐MSCs to enhance spatial learning and memory ability in SAMP8 mice (Figure 6). Therefore, HGF underpins the reparative functions of hUC‐MSCs and can replace hUC‐MSCs to improve cognitive ability in AD mice and restore the function of neurons to a degree.

HGF is a pleiotropic cytokine primarily made by cells of mesenchymal origin. Originally described as a major mitogen for hepatocytes,^[^ ^47^ ^]^ HGF has been identified in multiple different tissues including the CNS.^[^ ^48^ ^]^ HGF is relatively stable in blood and crosses the blood brain barrier by a saturable transport system. At least a third of the HGF reaching the cerebral circulation can enter the parenchyma of the brain. Thus, the periphery can provide a source of HGF that could be important in the CNS, particularly when additional trophic support is needed.^[^ ^49^ ^]^ The biological effects of HGF are primarily mediated by the tyrosine kinase transmembrane receptor cMet.^[^ ^50^ ^]^ During development, cMet is expressed in several different tissues including the CNS^[^ ^51^ ^]^ and is able to mediate all the known effects of HGF.^[^ ^26^ ^]^ Here, we used the HGF R/c‐Met antibody (cMet Ab) and certified that c‐Met could mediate the function of HGF for restoring neurological damage in vitro (Figure 7 and Figure S11, Supporting Information); more importantly, both MSC and HGF treatments could lower OA‐induced tau phosphorylation at Ser396, Thr212, and Ser214 compared to that of OA‐treated alone to different degrees, but the suppression of tau phosphorylation by MSC and HGF was partly restricted after the addition of c‐Met Ab (Figure 8D–I).

To further explore the mechanism of hUC‐MSCs in HGF‐mediated recovery of neurological damages, we used an exploratory hippocampal tissue‐based mRNA microarray to analyze and search potential pathways. By significant analysis of microarrays (SAM) of gene differential expression, 19 different genes were obtained and selected (Figure 8A); then, KEGG pathway analysis was used to evaluated the enriched pathways for the representative profiles of genes involved in signal transduction pathways (Figure 8B). Pathway analysis further certified that more genes were related with PI3K‐Akt signaling pathway (Figure 8C). PI3K is a key enzyme in downstream signaling through the AKT pathway, which has been implicated in memory.^[^ ^52^ ^]^ The phosphorylation of AKT was decreased in SAMP8 mice compared with that in SAMR1 mice.^[^ ^53^ ^]^ Activation of AKT, which inhibits GSK3*β*, is one of the most well characterized cell survival signaling pathways.^[^ ^27^ ^]^ So, we focused on the AKT‐GSK3*β* pathway in this study and verified it in vitro and in vivo. In an OA‐treated SH‐SY5Y cell model, p‐AKT (Ser 473) and p‐GSK3*β* (Ser 9) were decreased in OA‐treated SH‐SY5Y cells compared to that of the control group. Both CM and HGF significantly increased the immunoblot reactivity of p‐AKT and p‐GSK3*β* in OA‐treated cells compared to that in OA‐treated cells alone. The positive effects of phosphorylation of AKT and GSK3*β* by CM and HGF were partly restricted after the addition of c‐Met Ab (Figure 8G–I). Phosphorylation of AKT (Ser 473) and GSK3*β* (Ser 9) was decreased in PBS‐treated SAMP8 mice compared to that in SAMR1 mice which was consistent with previous studies,^[^ ^53^ ^]^ and both hUC‐MSC and HGF treatment could restore the level of p‐AKT and p‐GSK3*β* in SAMP8 mice which may decrease the hyperphosphorylation of tau protein at Ser396, Thr181, and Ser404 (Figure 8J–L). Hence, the mechanism through which hUC‐MSC and HGF overcame tau hyperphosphorylation may involve the AKT‐GSK3*β* signaling pathway.

Although we have demonstrated that HGF secreted from hUC‐MSCs could mediate hUC‐MSC‐modulated recovery of neurological damage and cognitive function at least to some extent, we still believe that in addition to HGF, there may be other bioactive components that mediate hUC‐MSCs to play important roles. In fact, we are also concerned about the potential therapeutic effect of other highly expressed factors on AD and other cognitive dysfunction (Figure 4A,B). For example, among the most strongly expressed 18 factors (Figure 4B), there are some important inflammatory factors involved in the immune response, including IL‐6, MCP3, MCP2, LAP, ENA‐78, which are involved in the inflammation of the nervous system and play crucial roles, but little is known about their roles in the occurrence and development of AD. In our research, we screened through high‐throughput screening and found that IL‐6 is the strongest secreted factor expressed by hUC‐MSCs (Figure 4A,B), and verified that IL‐6 could repair and protect damaged neural cells which effect was second only to that of HGF (Figure S5, Supporting Information); and some other studies have confirmed that IL‐6 could regulate the adenosine A1R and A2aR expression and stimulate ganglion retinal cells to produce neuroprotective factors such as BDNF, thereby exerting a neuroprotective effect;^[^ ^42^ ^]^ however, other studies have shown that IL‐6 could increase neurotoxicity caused by A*β*.^[^ ^54^ ^]^ Therefore, the regulation of inflammatory factors on the AD brain may be more complicated, and only through careful design and in‐depth research under specific time and space conditions can we further understand its function. In addition, there are some factors with the moderate expression, such as PDGF‐AA, ALCAM, FGF7, IGFBP6, FASLG, etc; although their expression intensity is relatively low, their roles in hUC‐MSC‐treatment of AD also need to be explored. Therefore, a further excavation on the exact roles of different core factors of hUC‐MSCs might be the key to delineate the therapeutic value of hUC‐MSCs in AD. AD is a complex disease characterized by multi‐pathological features, in which both A*β* plaques and NFTs are neurotoxic, and optimal approaches for AD treatment might be able to target the most toxic species of both A*β* and tau concurrently. Our ultimate goal is to develop various HGF combination therapies (dependent or independent of clinical‐grade hUC‐MSCs) for AD patients, including targeting of the A*β* and tau pathological changes and optimizing the existing regulatory methods for the inflammatory environment, to finally achieve a comprehensive therapy for AD; but all these speculations require further digging and verification, and we plan to explore more in future research.

## 4 Conclusion

To our knowledge, this is the first study to use SAMP8 mice (a senescence‐accelerated mouse model of AD) to explore the effects and mechanisms of clinical‐grade hUC‐MSCs on the recovery of cognitive ability. Collectively, the data demonstrated that the core functional factor HGF secreted from hUC‐MSCs plays important roles in hUC‐MSC‐modulated recovery of damaged neural cells by down‐regulating hyperphosphorylated tau, improving the NFT, reversing spine loss, and promoting synaptic plasticity in the hippocampus of AD mice, which are all closely associated with memory deficits. Mechanistically, the structural and functional recovery, as well as cognitive enhancements elicited by exposure to hUC‐MSCs, were at least partially mediated by HGF in the AD hippocampus through the activation of cMet‐AKT‐GSK3*β* signaling pathway. Hence, we suggest that hUC‐MSC treatment could be a promising and effective neuroprotective candidate to prevent AD and other progressive age‐related neurodegenerative diseases. Crucially, these findings raise the possibility that HGF may be used in isolation or in combination with hUC‐MSCs for AD treatment.

## 5 Experimental Section

##### Clinical‐Rade hUC‐MSCs and Condition Medium Collection

All procedures involving human subjects in this study were approved by Ethics Committee at the Third Affiliated Hospital of Sun Yat‐sen University (Approval number: 2017–19); and all the patients gave their written informed consent to participate. Clinical‐grade hUC‐MSCs were used in this study which were greatly optimized in a previous research.^[^ ^14^ ^]^ The entire link including isolation, cultivation, identification, quality control, and storage was conformed to the quality standards. The generation time of hUC‐MSCs used in this study was severely restricted from passage 3 (P3) to passage 5 (P5).

hUC‐MSCs were seeded at initial density of 1 × 10^4^ cells cm^−2^ in 10 cm dishes, cultured for 24 h, and the medium replaced with 8 mL of *α*‐MEM for additional 48 h, CM was centrifuged (2500 rpm for 5 min) to remove cell debris and used for experiments.

##### Neural Cell Culture and Drug Treatment

Primary hippocampal neurons were prepared from embryonic day 18 rat embryos and plated on 25‐mm coverslips or 35‐mm glass bottom dishes pretreated with 0.1 mg mL^−1^ poly‐d‐lysine (Sigma) at a density of ≈350 000 cells per dish. Neurons were plated and maintained in Neurobasal medium supplemented with B27, N2, and GlutaMax (Invitrogen)in a humidified incubator at 37 °C with 5% CO_2_. SH‐SY5Y cells (purchased from the Shanghai Institutes for Biological Sciences, China) were supplied with complete in Dulbecco's modified Eagle's medium (DMEM) supplemented with 10% fetal bovine serum, 2 mm glutamine.^[^ ^55^ ^]^ To establish the in vitro model of tauopathy which was hyperphosphorylation of tau, SH‐SY5Y cells were induced by incubation with okadaic acid (OA, Sigma) at a final concentration of 20 nm for 24 h as a previous work,^[^ ^19^ ^]^ and neurons were induced by incubation with OA at a final concentration of 10 nm for 4 h, according to the different sensitivity to OA. For cell treatment, OA‐damaged SH‐SY5Y cells or neurons were replaced with CM or HGF, while Con and OA‐damaged groups were replaced with *α*‐MEM. To neutralize the function of HGF in CM and block the function of c‐Met of neural cells, HGF antibody (HGF Ab) (R&D) and HGF R/c‐Met antibody (R&D) were used in the experiment.

Neural stem cells (neurospheres) cultures were prepared from newborn rat cortex and grown in serum‐free medium containing EGF and bFGF (both 20 ng mL^−1^).

##### Animals and Treatment

The senescence accelerated mouse prone 8 (SAMP8), and senescence accelerated mouse resistant 1 (SAMR1) mice were purchased from Peking University Health Science Center. 4‐month‐old male SAMP8 and SAMR1 mice were used in this study. The vehicle‐treated mice were received with 500 µL phosphate buffered saline (PBS). the SAMP8 mice was treated with hUC‐MSCs (also called MSC^WT^ in Figure 6), MSC^Con^, or MSC^ShHGF^ at a dose of 5 × 10^6^ cells in 500 µL PBS by intraperitoneal administration once a week for 8 weeks; or HGF treatment at a dose of 100 ng in 500 µL PBS in each mouse by tail intravenous administration; SAMR1 mice were used as normal group. A previous research found that the concentration of human HGF in the tail vein blood of SAMP8 mice could reach at least 50 ng mL^−1^ 1 day after intraperitoneal injection of hUC‐MSCs (5 × 10^6^ cells) (data not shown). The average body weight of 4‐month‐old SAMP8 mice was about 30 g, and their whole blood was about 2 mL. In order to achieve the dose of human HGF in vivo after hUC‐MSCs injection, HGF alone was used at a dose of 100 ng. All animals received care according to the Guide for the Care and Use of Laboratory Animals. The protocol was approved by the Committee on the Ethics of Animal Experiments of Academy of Military Medical Sciences.

##### Microarray Analysis: Human Cytokine Antibody Array

To determine the secretory profile of the CM generated from hUC‐MSCs, antibody arrays (RayBio human cytokine antibody G‐Series 2000; Ray Biotech, Inc., Norcross, GA) were used in this experiment. Signal intensity values representing detected cytokines were subtracted from the background and normalized to positive controls on the same membrane. Experimental steps and analyses were conducted according to the manufacturer's instructions. Signal intensity values of each cytokine were presented as mean ± standard deviation (SD).

##### Mouse mRNA Microarray

For signal pathway research, mRNA microarray was used to analyze and verify the probable pathway. Total RNAs was quantified by the NanoDrop ND‐2000 (Thermo Fisher Scientific) and the RNA integrity was assessed using the Agilent Bioanalyzer 2100 (Agilent Technologies). The sample labeling, microarray hybridization and washing were performed based on the manufacturer's standard protocols. After washing, the arrays were scanned by the Agilent Scanner G2505C (Agilent Technologies). Raw data were extracted using Feature Extraction (version10.7.1.1; Agilent Technologies). Next, quantile normalization and subsequent data processing were done using Genespring software (version 13.1; Agilent Technologies). The microarray profiling, differential gene expression analysis and pathway analysis were conducted in the laboratory of the OE Biotech Company (Shanghai, China).

##### Statistical Analysis

The intensity of IHC staining, were analyzed using Image‐Pro Plus 6.0 software (Media Cybernetics, Inc., Maryland). Statistical analyses were performed with GraphPad Prism 8.0 software (GraphPad Software, San Diego, CA, USA) and expressed as mean plus or minus standard error of the mean. Multiple groups were compared using one‐way analysis of variance (ANOVA) followed by the Tukey test for post hoc comparisons based on single factor experiments. In behavioral study of MWM task, the data of the training trials were analyzed using a two‐way ANOVA with days as repeated measures factor and treatments as between subjects’ factor. For comparisons of the mean between two groups, statistical analysis was performed by applying Student's *t* tests. Statistical significance was set at *P* < 0.05. All data were shown as means ± SEM or means ± SD.

## Conflict of Interest

The authors declare no conflict of interest.

## Supporting information

Supporting Information
