# Supplementary material for: HGF Mediates Clinical‐Grade Human Umbilical Cord‐Derived Mesenchymal Stem Cells Improved Functional Recovery in a Senescence‐Accelerated Mouse Model of Alzheimer's Disease
Source: Adv Sci (Weinh). 2020 Jul 6;7(17):1903809. doi: 10.1002/advs.201903809 (PMC7507104; doi:10.1002/advs.201903809)
Supplement: Supplementary file 1 — Supporting Information [file ADVS-7-1903809-s001.pdf]

Supplemental Methods

Water maze performance: The Morris water maze apparatus was previously described.[1] Mice were trained once a day over four consecutive days. In each trial the mouse swam until it found the platform, or after 60 s it was guided to the platform; the mouse was then placed on the platform for 15 s before being picked up. At the end of the testing period, place navigation (60 s) was performed. The spatial probe was done 5 days after navigation test. Removed the platform and recorded the crossing number of original platform position in 60 s. Data were collected and analyzed using Water Maze system (Biobserve).

Open field test: Open-field (OF) test apparatus was constructed of white plywood (50×50×25 cm). Red lines were drawn on dividing the floor into 25-cm squares. Behavior was scored with SMART® ver. 3.0 software, and each trial was recorded for later analysis, utilizing a camera fixed to the ceiling at a height of 2.1 m situated above the apparatus. Mice were placed at the center, or at one of the four corners, of the open field and allowed to explore the apparatus for 5 min. After the 5-mintest, the mice were returned to their home cages and the open field was cleaned with 70% ethyl alcohol and allowed to dry between tests. To assess the animal's habituation process to the novelty of the arena, the mice were exposed to the apparatus for 5 min on two consecutive days. The behaviors scored included line crossing, center entries, center stay duration, rearing, defecation, and urination. Each animal was then given a score for total locomotor activity, which was calculated as the sum of total distance, line crosses, and number of rears.[2]

Contextual Fear Conditioning: In the fear conditioning experiment, the training, context and alter context phases of the fear conditioning (FC) task were administered over a period of 3 day. (*i*) Familiarity and Modeling: Place the mice individually in the fear box, adjust for 2 minutes, record the freezing time (the data as the baseline), then give the single frequency of sound signal (4.5 kHz, 60 dB, 30 s), then give an inescapable foot shock (0.5 mA, 2 s) with the sound and shock simultaneously ending; after interval 1 minute, start the next cycle of sound stimulation and electric shock, with a total of 10

cycles. At the end of each experiment, clean the bottom of the box and the wall with 75% alcohol to remove any residual odor. (*ii*) Context text phase: 24 hours later, give no sound stimulation or electrical stimulation, place each mouse in the fear box and record the freezing time (Freezing) within 5 minutes. (*ii*) Altercontext text phase: after 24 hours, change the environment inside the fear box (change the metal fence at the bottom of the box to a plastic plate, change the color of the box, spray a small amount of lemonade), and then give a single frequency of sound signal (4.5 kHz, 60 dB, 30 s), and record the freezing time (Freezing) within 5 minutes.

Extracellular electrophysiology: Mice were sacrificed by decapitation after anesthesia using 1% pentobarbital sodium (50 mg kg^{-1}). Brain was removed carefully and immediately soaked in ice-cold incubation solution (ACSF: 1L H_2O; NaCl, 6.838g; KCl, 0.268g; $KH_2PO_4 \cdot 2H_2O$, 0.187g; $CaCl_2 \cdot 2H_2O$, 0.3675g; $MgCl_2 \cdot 6H_2O$, 0.244g; $NaHCO_3$, 2.1g; and glucose, 1.98g) which had been well saturated through exposure to 95% O_2 and 5% CO_2 for 1 h at room temperature. Brain slices (400 μm thick) were prepared using vibrating tissue slicer (Microslicer™DTK-1000; DOSAKA), and soaked in oxygenated ACSF for 1h at 32°C. The hippocampal regions were resected and placed on the center on the MED-P515A probe (Alpha MED Scientific) with 64 embedded recording electrodes. After positioning of the slice on MED probe, microscopic photograph was taken. MED probe was placed on the MED connector and incubation solution oxygenated with 95% O_2 and 5% CO_2 was continuously infused at the rate of 2 mL min^{-1} (at 32˚C). The field excitatory postsynaptic potentials (fEPSP) were recorded using a MED64 multichannel recording system (Mobius Win7 0.5.0, WitWerx Inc.), and the data were collected from the dendritic layer of the CA1 region at a sampling rate of 10 kHz. For each slice, the baseline stimulus intensity was set at the level that elicited ~50% of the maximum fEPSP response, which was determined according to the input–output curve. Long-term potentiation (LTP) was induced by 3 trains of HFS (100 Hz for 1 s delivered 30 s apart). LTP was calculated as the mean percentage change in the amplitude of the population spike after high-frequency stimulation relative to its basal amplitude.[3]

Histology and Immunohistochemistry: Mice were perfused transcardially with 50ml PBS, followed by 50ml of 4% paraformaldehyde and their brains were removed and post-fixed overnight in 4% PFA. Each brain was embedded in paraffin and 5μm-thick coronal sections. Standard histological Hematoxylin-Eosin staining procedure was performed. Tissue sections were incubated overnight with primary antibodies: rabbit monoclonal anti-Phospho-Tau (Thr181), rabbit monoclonal anti-BACE1 (D10E5, Cell Signaling Technology), mouse monoclonal anti-Presenilin 1 (PS-1, APS18, Abcam); rabbit polyclonal anti-Nestin, rabbit monoclonal anti-Sox2, rabbit polyclonal anti-Phospho-GSK3β (Tyr216) (Abcam), and staining was revealed using biotinylated secondary antibodies and the ABC kit (Vector with diaminobenzidine (DAB; Sigma-Aldrich). Individual cell numbers were quantified by pTau (Thr181), pGSK3β (Tyr216), Nestin and Sox2 and quantified as the mean signal intensity using Image-Pro Plus 6.0 8 software (Media Cybernetics, Inc., Maryland).

Immunofluorescence staining: Each brain was embedded in OCT 8 μm-thick coronal sections and cell cultures pre-incubated in 10% normal goat or donkey serum, Triton 0.3% in PBS for 30 min. Tissue sections or cell cultures were incubated overnight at 4°C with the following antibodies: rabbit polyclonal anti-Nestin (Abcam), rabbit monoclonal anti-β-Tubulin III (Tuj-1, Sigma), mouse monoclonal anti-α-tubulin (Cell Signaling Technology); rabbit polyclonal anti-Iba-1 (Wako), mouse monoclonal anti-ED1(CD68, Abcam), mouse monoclonal anti-GFAP (GA5, Cell Signaling Technology). Secondary antibodies (1:100, Jackson ImmunoResearch Laboratories, PA). Nuclei were visualized using 2 μM DAPI (Sigma). F-actin was labeled with rhodamine phalloidin (Invitrogen), and the dendritic spine was labeled with the lipophilic tracer 1,1'-dioctadecyl-3,3,3'3,3'-tetramethylindocarbocyanine perchlorate (Dil, Molecular Probe). Mitochondrial function was evaluated with the cationic dye 5,5',6,6'-tetrachloro-1,1',3,3'-tetraethylbenzimidazolylcarbocyanine iodide (JC-1, Molecular Probe). Thioflavin-S (Sigma) staining was used to observe the NFT-like pathologies.

Western blot analysis: Mice hippocampal were dissected after sacrificed, snap frozen and lysed in RIPA buffer (50 mM Tris-HCl, pH 7.5, 150 mM NaCl, 1% NP-40, 0.5%

sodium deoxycholate, 0.1% SDS), protease inhibitor cocktail tablets (Roche, Germany) and Phosphatase inhibitor cocktail tablets (Roche, Germany). Western blots were performed and analyzed as previously described.[4] Antibodies used for Western blots were: Sox2, Nestin (Abcam); pTau (Ser396, Thr212, Thr214, Invitrogen), pTau (Thr181, Ser404, Cell Signaling Technology); p-AKT (Ser 473), p-GSK3β (Ser 9), GAPDH (Cell Signaling Technology).

Electron microscopy: Mice were perfused with 2.5% glutaraldehyde. Hippocampal were isolated, dorsally sectioned with a vibratome at 400 μm and then resected to 100μm. Hippocampal were post-fixed in 1% OsO4 for 1 hour, dehydrated and embedded in epoxy resin. Electron micrographs (100 μm^2 CA1 and DG area at 8,000X) were made of hippocampal sections with an electron microscope (Hitachi H-7650).

[1] M. Costa-Mattioli, D. Gobert, H. Harding, B. Herdy, M. Azzi, M. Bruno, M. Bidinosti, C. Ben Mamou, E. Marcinkiewicz, M. Yoshida, H. Imataka, A.C. Cuello, N. Seidah, W. Sossin, J.C. Lacaille, D. Ron, K. Nader, N. Sonenberg, *Nature* **2005**, *436*, 1166.

[2] M.G. Morgese, P. Tucci, E. Mhillaj, M. Bove, S. Schiavone, L. Trabace, V. Cuomo, *Mol Neurobiol* **2017**, *54*, 2079.

[3] A.K. Fu, K.W. Hung, H. Huang, S. Gu, Y. Shen, E.Y. Cheng, F.C. Ip, X. Huang, W.Y. Fu, N.Y. Ip, *Proc Natl Acad Sci U S A* **2014**, *111*, 9959.

[4] Y.L. Jia, L. Shi, J.N. Zhou, C.J. Fu, L. Chen, H.F. Yuan, Y.F. Wang, X.L. Yan, Y.C. Xu, Q. Zeng, W. Yue, X.T. Pei, *Hepatology* **2011**, *54*, 1808.

Supplemental Figures and Legends

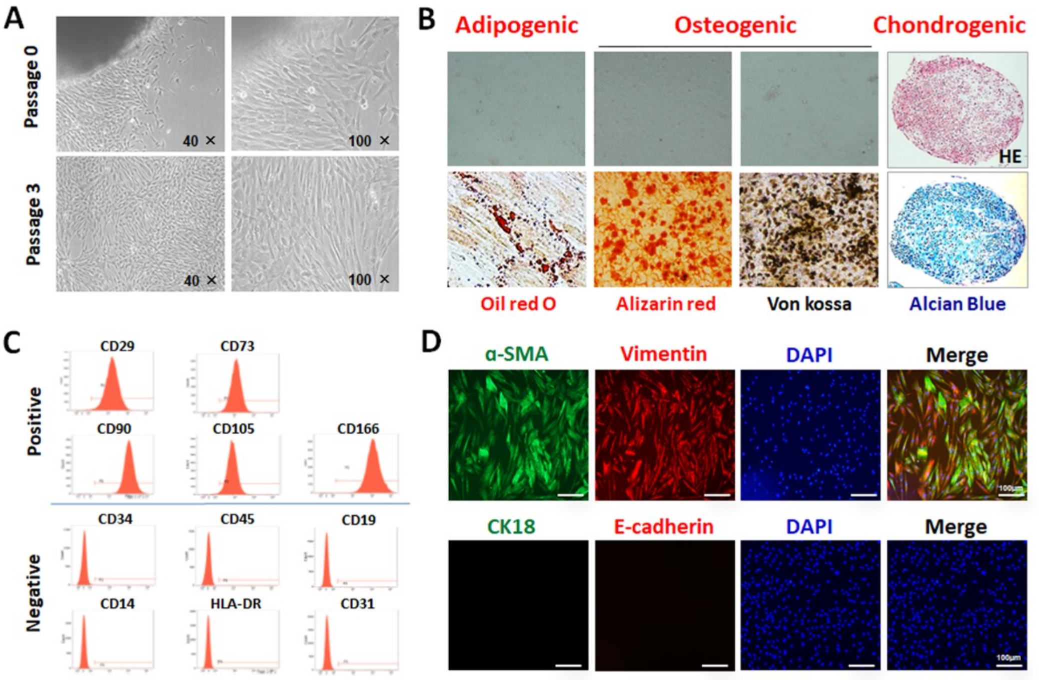

Figure S1. Characteristics and differentiation potential of clinical-grade hUC-MSCs derived from primary umbilical cord. (A) Representative optical morphological images of primary clinical-grade hUC-MSCs of P0 and P3 derived from optimized tissue blocks (Magnification: ×40 and ×100) **(B)** Differentiation potential of hUC-MSCs into mesodermal lineages. Representative images of hUC-MSCs differentiated into adipocytes, osteocytes and chondrocytes are shown as indicated. Fat droplets were stained with Oil red O. Calcium phosphate deposits were stained with Alizarin Red, and lots of calcium deposition in the extracellular matrix, which were verified by von Kossa staining. Proteoglycans with Alcian Blue. **(C)** Flow cytometric analysis showed hUC-MSCs were positive for mesenchymal lineage markers (CD29, CD73, CD90, CD105 and CD166), negative for hematopoietic and endothelial markers (CD34, CD45, CD19, CD14, and CD31), and negative for HLA-DR. **(D)** Immunofluorescence staining of hUC-MSCs showed they were positive for mesenchymal markers of α-SMA (Green) and Vimentin (Red), and negative for epithelial markers of CK18 and E-cadherin. (Scale bar=100 μm).

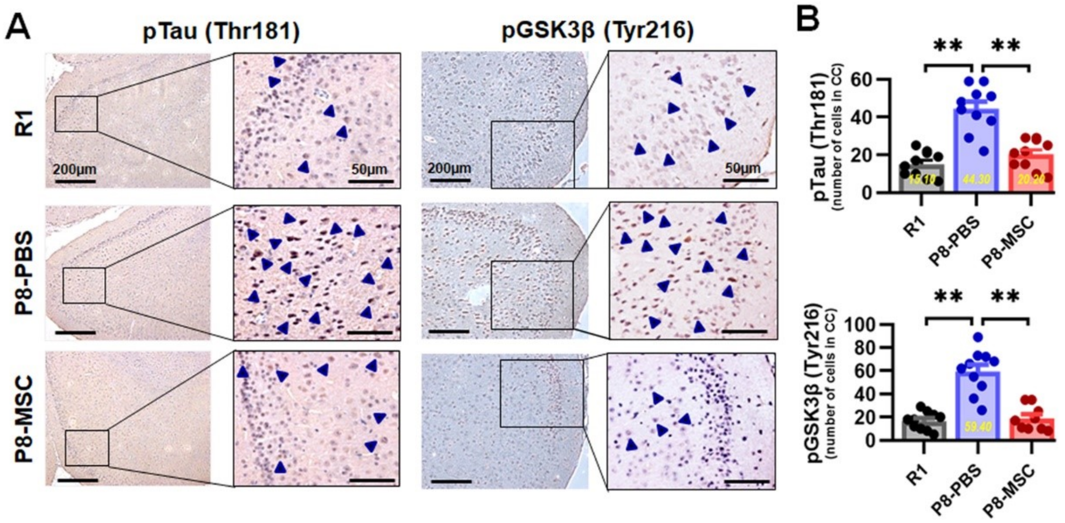

Figure S2. hUC-MSCs regulated the expression of pTau and pGSK3β in cerebral cortex of SAMP8 mice. (A) Representative images of cerebral cortex showing the expressions of pTau (Thr181) and pGSK3β (Tyr216) in R1, P8-PBS and P8-MSC mice by Immunohistochemical method (Scale bar=200 μm). Squares in higher-magnification inserts indicate the protein positive cells with arrowheads-labeled individual cell (Scale bar=50 μm). **(B)** Quantification of pTau (Thr181) and pGSK3β (Tyr216) in cerebral cortex region of the R1, P8-PBS and P8-MSC groups (n=9-10/group; all data shown as mean ± SEM, *P < 0.05, **P < 0.01). CC: cerebral cortex.

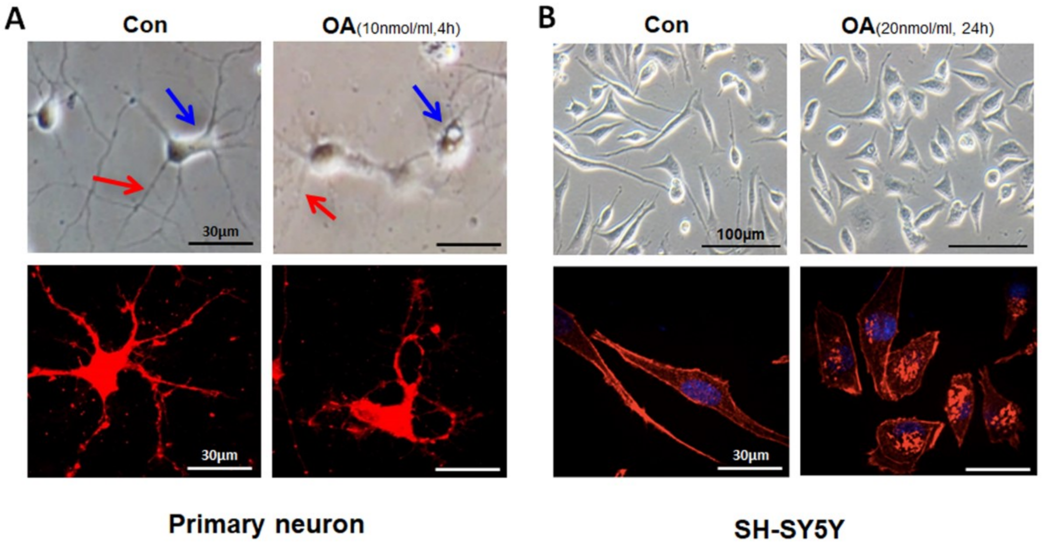

Figure S3. Establishing AD cell model *in vitro*. The primary neurons were induced by incubation with OA at final concentration of 10 nM for 4 hours **(A)** and SH-SY5Y cells were induced by incubation with OA at final concentration of 20 nM for 24 hours **(B)**. Magnification: optical morphological images of neuron (Scale bar=30 μm) and SH-SY5Y cells (Scale bar=100 μm), immunofluorescence images (Scale bar=30 μm).

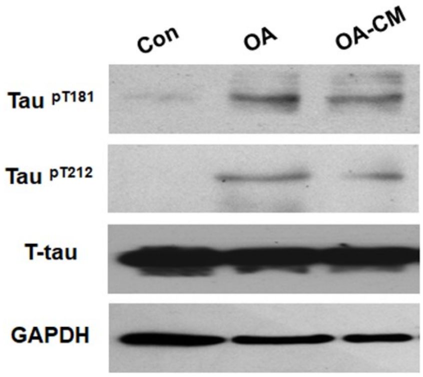

Figure S4. hUC-MSCs could in part inhibit the tau hyperphosphorylation in OA-induced SH-SY5Y cell model. Representative western blotting results showing the expression of phospho-Tau (Thr212 and Ser396) of SH-SY5Y cells in Con, OA and OA-CM groups.

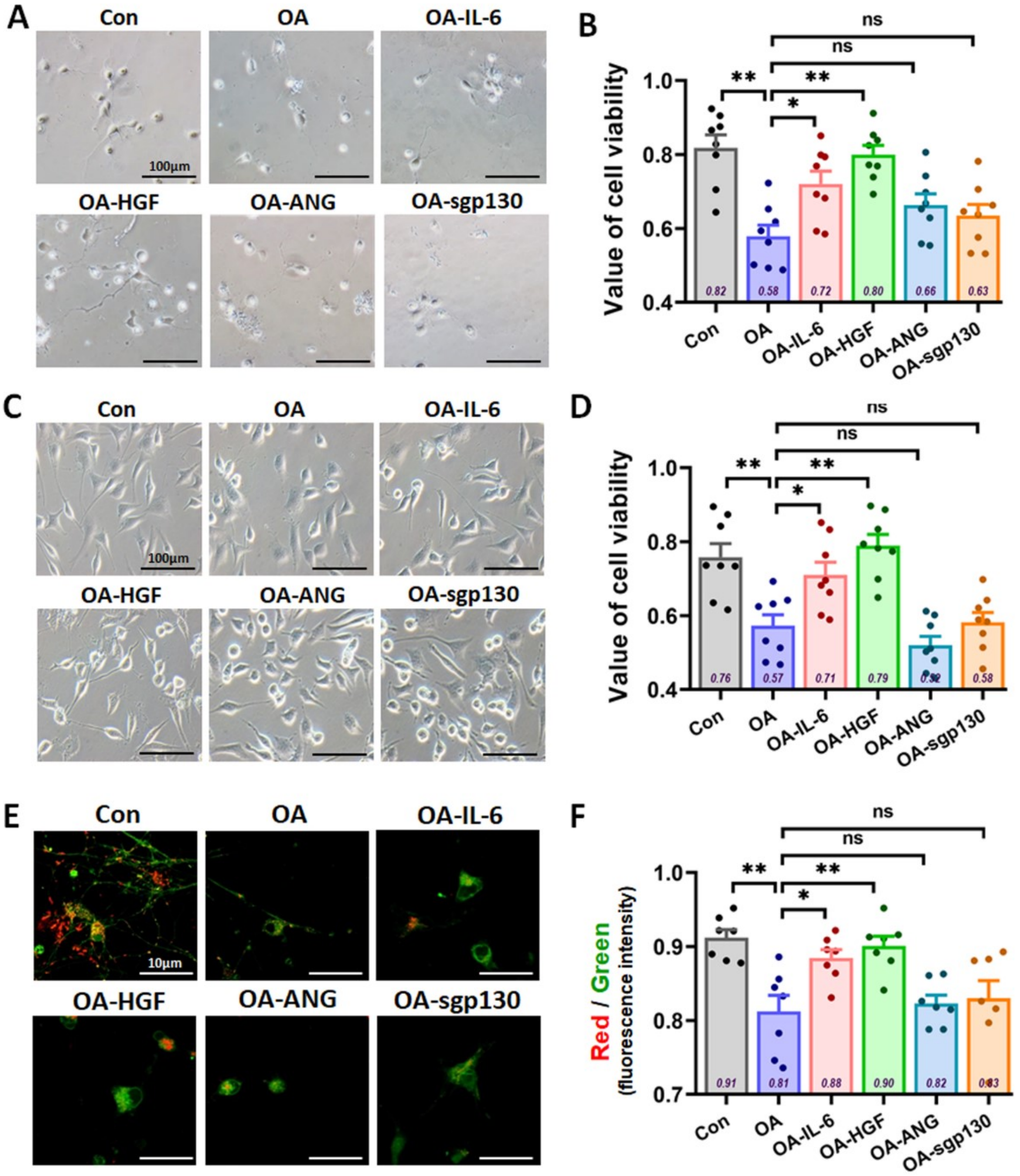

Figure S5. Screening four cytokines using AD cell model *in vitro*. Representative optical morphological images of primary neurons **(A)** and SH-SY5Y cells **(C)** using OA-induced damage model. Groups including Con, OA, and adding *in turn* IL-6, HGF, ANG, GRO (Scale bar=100 μm). **(B, D)** Value of cell vitality in different group with or without cytokine in OA-induced neurons damage model **(B)** and SH-SY5Y cell damage model **(D)**. **(E)** Representative images of JC-1-labeled neurons in different cytokines treatments (Scale bar=10 μm). **(F)** The quantification of Red/Green fluorescence intensity in the six groups (All data shown as mean ± SEM, $^*P < 0.05$, $^{**}P < 0.01$).

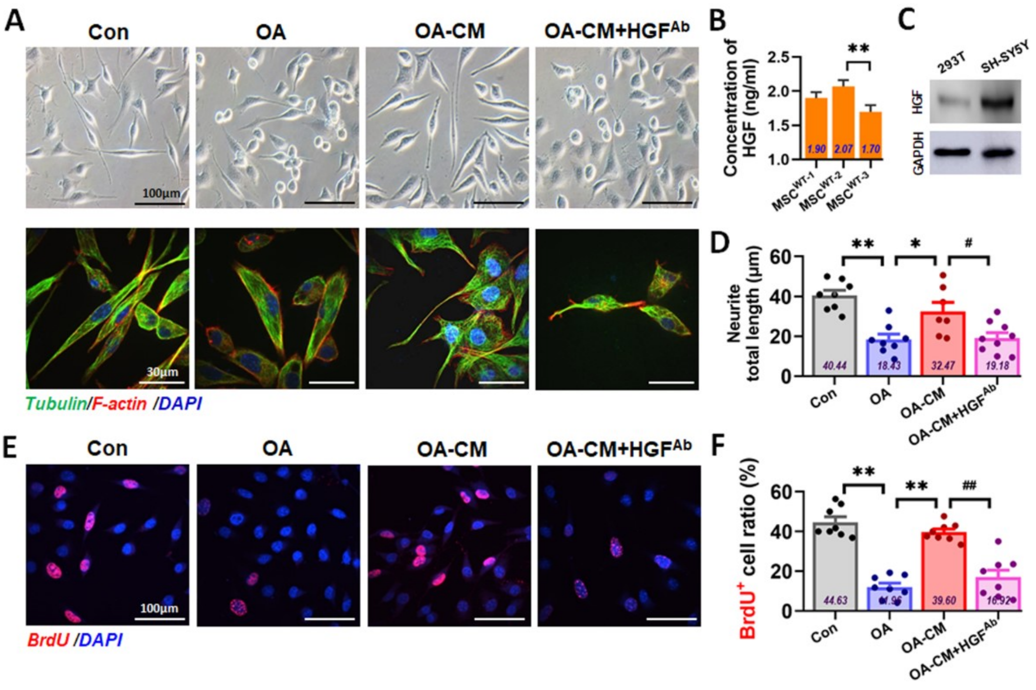

Figure S6. Inhibition of HGF signaling weakened the capacity of hUC-MSCs to induce functional recovery and reverses. (A) Representative morphological images of SH-SY5Y cells observed by light microscope (*Upper*, Scale bar=100 μm) and confocal laser scanning (*Lower*, Scale bar=30 μm) in Con, OA, OA-CM, OA-CM+HGF-Ab groups. **(D)** The quantification of the neurite mean length of SH-SY5Y cells in the four groups (n= 7-9/group; data shown as mean ± SEM). **(B)** The concentration of HGF in hUC-MSC-conditional medium (CM) from three different umbilical cords (UC1, 2, 3)-derived hUC-MSCs was detected by ELISA (n= 3/group; data shown as mean ± SD). **(C)** The expression level of HGF in SH-SY5Y cells was detected by Western blotting, and 293T cells was used as positive control. **(E)** Confocal laser scanning images of Brdu-labeled SH-SY5Y cells in Con, OA, OA-CM and OA-CM+HGF-Ab groups (Scale bar=100 μm). **(F)** The quantification of BrdU-positive cells in the four groups (n=8/group; data shown as mean ± SEM). *P < 0.05, **P < 0.01; $^{\#}$P < 0.05, $^{\#\#}$P <0.01.

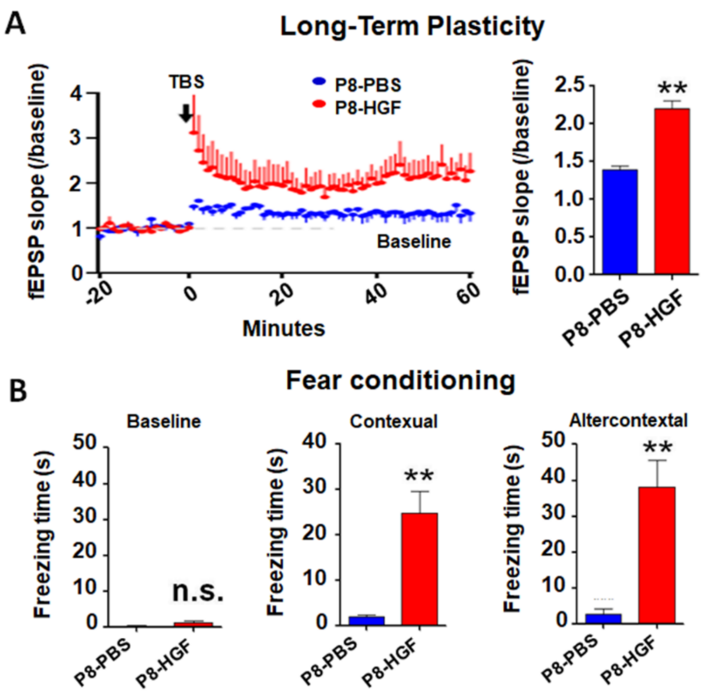

Figure S7. HGF enhanced the hippocampal synaptic plasticity and fear conditioning associated-memory in SAMP8 mice. (A) HGF rescued LTP impairment in P8 mice. LTP in the hippocampal CA1 region was induced by high-frequency stimulation (HFS) (n=4-7/group). *Left:* Averaged slopes of baseline normalized fEPSP. *Right:* Quantification of mean fEPSP slopes during the last 10 min of the recording after LTP induction. **(B)** Experiment of the fear conditioning with the chronological order for the freezing time of freezing behavior across baseline, the context test phase and the altercontext test phase. (n=6-8 mice/group; all data shown as mean ± SEM, **P <0.01).

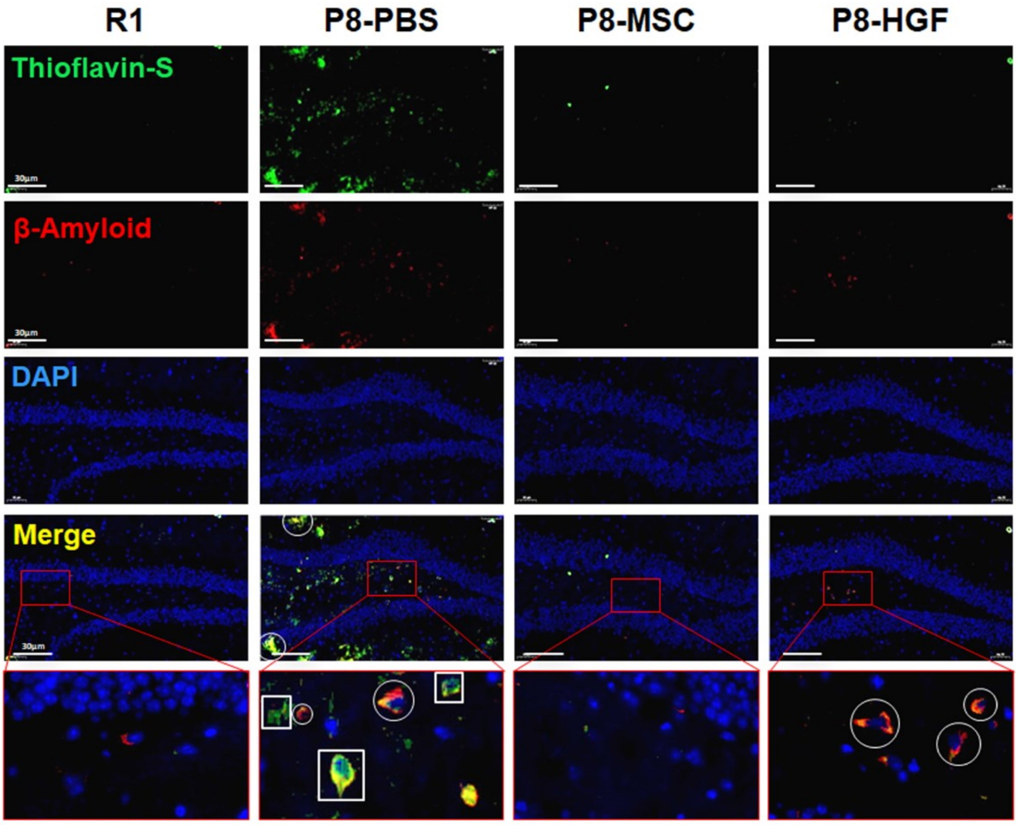

Figure S8. HGF regulated the expression of AD-related key proteins. Representative immunohistochemical images of hippocampal DG region showing the distribution of neurofibrillary tangles (NFT) and senile plaques (SP)-like pathologies in R1, P8-PBS, P8-MSC and P8-HGF mice. Red boxes in higher-magnification inserts indicate the NF-like pathologies with squares-labeled individual cell and SP-like pathologies with circles-labeled individual cell (Scale bar=100 μm). Thioflavin-S: Green, β-Amyloid: Red, DAPI: Blue.

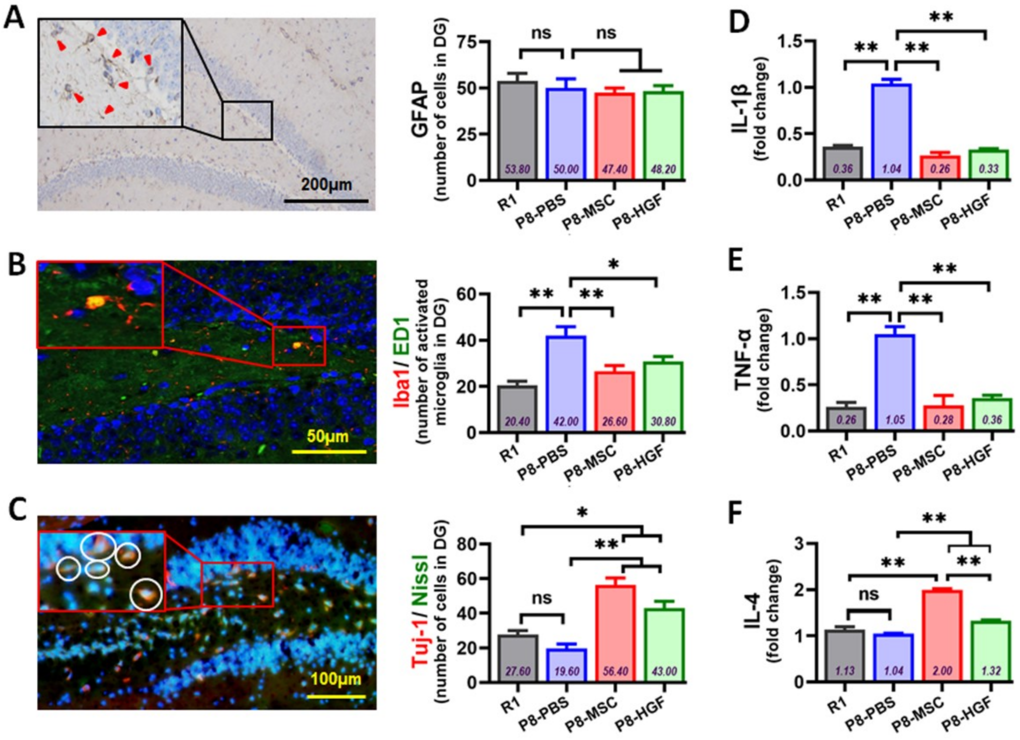

Figure S9. Evaluation of reversal effects of hUC-MSCs and HGF on neuroinflammation and neuronal damages in SAMP8 mouse brain. (A) Representative images of hippocampal dentate gyrus (DG) showing the expression of glial fibrillary acidic protein (GFAP, a marker of astrocyte activation) in R1, P8-PBS, P8-HGF, and P8-MSC mice by Immunohistochemical method. *Right*: quantification of GFAP in DG region of the R1, P8-PBS, P8-HGF, and P8-MSC groups. **(B)** Representative images of activated microglia (labeled with Iba1^{+}/ ED1^{+}) in DG region by Immunofluorescence method (*Left*), and quantification of Iba1 and ED1 microglia (*Right*) of the R1, P8-PBS, P8-HGF, and P8-MSC groups. **(C)** Representative images of neurons (labeled with Tuj-1 and Nissl) in DG region by Immunofluorescence method (*Left*), and quantification of Tuj-1^{+}/Nissl^{+} neurons (*Right*) of the R1, P8-PBS, P8-HGF, and P8-MSC groups. Squares in higher-magnification inserts indicate the protein positive cells with arrowheads-labeled individual cell. **(D-F)** The expressions of neuroinflammation related IL-1β, TNF-α and IL-4 in the hippocampus of the four mouse groups were measured by qPCR; the expression of the different genes was normalized by β-actin. After hUC-MSCs or HGF treatment, there were significant decreases in RNA expression of both IL-1β **(D)** and TNF-α **(E)**, and increase in RNA expression of IL-4 **(F)**. All the data suggested that both hUC-MSCs and HGF produced sustained neuroprotective effects on SAMP8 mouse brain. (A-C: n=5/group; D-F: n=3/group; all data shown as mean ± SEM, *P < 0.05, **P < 0.01).

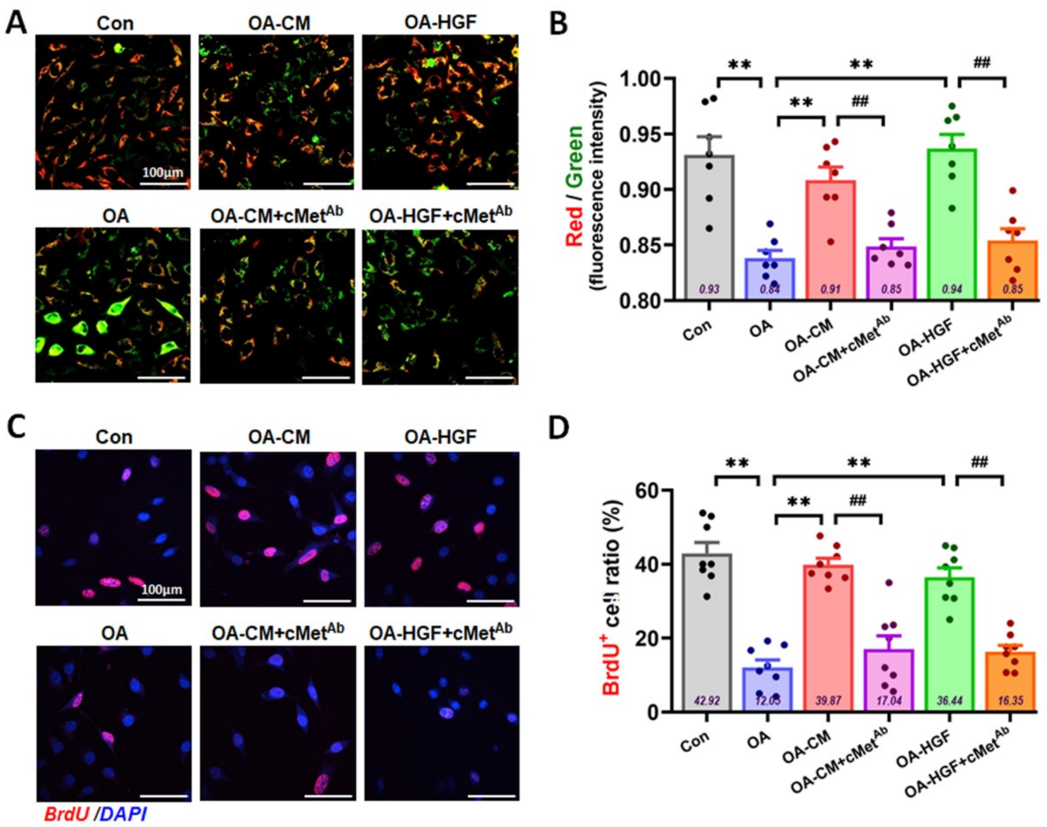

Figure S10. Blocking c-Met could restrain the capacity of HGF to recover the damaged SH-SY5Y cells. (A) Confocal laser scanning images of JC-1-labeled SH-SY5Y cells in Con, OA, OA-CM, OA-HGF, OA-HGF+cMet Ab and OA-HGF+cMet Ab groups. **(B)** The quantification of the Red/Green fluorescence intensity of the SH-SY5Y cells in the six groups. **(C)** Confocal laser scanning images of Brdu-labeled SH-SY5Y cells in different groups. **(D)** The quantification of BrdU-positive cells in the six groups. (n=7-8/group; all data shown as mean ± SEM, $^*P < 0.05$, $^{**}P < 0.01$; $^{\#\#}P < 0.01$).

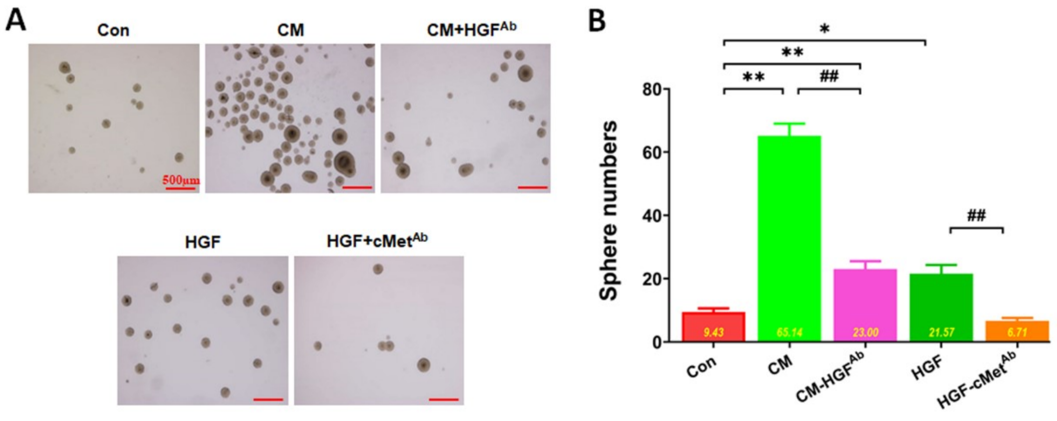

Figure S11. Both hUC-MSC-CM and HGF could promote the proliferation of NSCs *in vitro*. (A) Representative morphological images of NSC spheres in Con, CM, CM+HGF-Ab, HGF and HGF+cMet-Ab groups. In the culture system, CM significantly promoted neural sphere formation compared with the Con group, while HGF-Ab could inhibit the function of CM. HGF significantly promoted the neural sphere formation compared with the Con group, while cMet-Ab could dramatically inhibit the function of HGF (Scale bar=500 μm). **(B)** Quantification of neural sphere in different groups (n=7; all data shown as mean ± SEM, $^{*}P < 0.05$, $^{**}P < 0.01$; $^{\#\#}P < 0.01$).
